# RBM10 Deficiency Promotes Anti‐PD‐1 Resistance in LUAD via STING Alternative Splicing‐Driven CCL7 Signaling and Macrophage Polarization

**DOI:** 10.1002/advs.202522159

**Published:** 2026-06-22

**Authors:** Weitong Gao, Ruqiong Wang, Bo An, Lishuang Qi, Zihan Jing, Xingmei Ren, Yang Zhou, Mingjun Xu, Jiaojiao Li, Jie Liu, Liying Wang, Gang Xu, Rou Li, Dexin Jia, Yan Yu

**Affiliations:** ^1^ Department of Oncology Harbin Medical University Cancer Hospital Harbin Heilongjiang China; ^2^ College of Bioinformatics Science and Technology Harbin Medical University Harbin China; ^3^ Department of Radiation Oncology Shandong Provincial Hospital Affiliated to Shandong First Medical University Jinan China; ^4^ Department of Oncology Chaoyang Central Hospital of China Medical University Chaoyang Liaoning Province China; ^5^ Department of Respiratory and Critical Care Medicine Chongqing University Three Gorges Hospital School of Medicine Chongqing University Chongqing China; ^6^ China–Japan Friendship Hospital (Institute of Clinical Medical Sciences Beijing China; ^7^ China Chinese Academy of Medical Sciences & Peking Union Medical College Beijing China

**Keywords:** alternative splicing, immune checkpoint inhibitors, mitochondrial transfer, RNA‐binding motif 10, tumor‐associated macrophages

## Abstract

Although immune checkpoint inhibitors have improved outcomes in lung adenocarcinoma (LUAD), many patients still exhibit inadequate responses. The immunomodulatory functions of RNA‐binding motif (RBM) proteins remain poorly understood. Using in vivo and in vitro models of RBM10 deficiency combined with cytokine arrays, CLIP‐seq, RIP, and proteomics, we found that RBM10 deficiency promotes an immunosuppressive microenvironment, and targeting key chemokines restored anti‐PD‐1 efficacy in RBM10‐deficient LUAD models. RBM10 deficiency enhanced the polarization and recruitment of M2 tumor‐associated macrophages (TAMs), both in vitro and in vivo. Mechanistically, RBM10 loss disrupted STING exon 3 exclusion via alternative splicing and impaired QKI‐mediated stabilization of the STING‐E3(‐) isoform, shifting the splicing balance toward the STING‐E3(+) isoform and promoting CCL7 secretion. CCL7 acted through its receptor CCR2 on macrophages, driving M2 polarization and recruitment. This central pathway was further reinforced by a positive feedback loop wherein M2‐polarized TAMs transferred mitochondria to tumor cells, potentially contributing to mtDNA‐cGAS‐STING signaling and sustained CCL7 production. Therapeutically, CCL7/CCR2 blockade synergized with PD‐1 inhibition to promote tumor regression in RBM10‐deficient tumors. Collectively, RBM10 serves as a key immunoregulator in LUAD by modulating the STING‐CCL7‐CCR2 axis, and targeting the CCL7‐CCR2 axis represents a promising strategy to overcome anti‐PD‐1 resistance.

## Graphical abstract

RBM10 deficiency promotes anti‐PD‐1 resistance in lung adenocarcinoma by altering STING alternative splicing, which enhances CCL7 secretion and CCR2‐dependent M2 macrophage polarization. A positive feedback loop via mitochondrial transfer sustains this immunosuppression. Targeting the CCL7‐CCR2 axis synergizes with anti‐PD‐1 therapy, offering a promising strategy for RBM10‐low tumors.

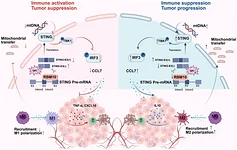

## 1 Background

Although chemotherapy and molecular‐targeted therapy remain standard treatments for lung adenocarcinoma (LUAD), their clinical benefits are limited, prompting increasing interest in immunotherapeutic strategies [1, 2]. FDA‐approved immune checkpoint inhibitors (ICIs) have achieved transformative success in LUAD, significantly improving objective response rates and overall survival (OS) [3, 4]. However, durable responses occur only in a subset of patients [5], underscoring the urgent need to elucidate mechanisms of immunosuppression within the tumor microenvironment (TME).

Tumor‐associated macrophages (TAMs) are the most abundant immune cells in the solid TME and exhibit remarkable heterogeneity and plasticity [6, 7]. TAMs are broadly categorized into classically activated M1 and alternatively activated M2 phenotypes. M2‐type TAMs, often induced by cytokines including macrophage colony‐stimulating factor (CSF), interleukin (IL)‐4, IL‐10, and IL‐13, facilitate tumor progression through extracellular matrix remodeling, angiogenesis and immune evasion [8]. In contrast, M1‐type TAMs, induced by interferon (IFN)‐γ and granulocyte macrophage colony stimulating factor (GM‐CSF), exhibit antitumor effects and correlate with better prognosis in LUAD patients [9, 10, 11]. Chemokines such as chemokine (C‐C motif) ligand 2 (CCL2), CCL5, and CCL7 play key roles in recruiting and functionally modulating TAMs [12, 13, 14]. Therefore, reprogramming TAMs by targeting chemokine signaling represents a promising T cell‐independent strategy to enhance ICIs efficacy, although optimal therapeutic combinations and patient selection require further investigation.

Alternative splicing (AS) affects over 95% of human protein‐coding genes, serving as a critical driver of proteomic diversity and evolutionary adaptation [15]. The dysregulation of AS drives oncogenesis by promoting the proliferation of tumor cells, facilitating metastasis and immune evasion, and conferring therapeutic resistance [16, 17]. RNA‐binding motif 10 (RBM10) is a tumor suppressor that regulates cancer‐related AS by binding specific pre‐mRNA sequences [18]. In LUAD, RBM10 modulates AS of key targets such as eukaryotic translation initiation factor 4H (EIF4H) and NUMB to inhibit tumor progression [19, 20]. For example, RBM10 inhibits the inclusion of exon 5 in EIF4H, which is critical for LUAD cell proliferation and survival. In hepatocellular carcinoma (HCC), RBM10 loss promotes CCL2 transcription and macrophage polarization by inhibiting NUMB exon 9 skipping and activating Notch signaling [21]. However, whether RBM10 regulates macrophage polarization in LUAD through AS‐dependent mechanisms remains poorly understood.

The stimulator of interferon genes (STING), an endoplasmic reticulum transmembrane protein, is a critical mediator of antitumor innate immunity [22]. Its activation promotes TANK‐binding kinase 1 (TBK1) and interferon regulatory factor 3 (IRF3) phosphorylation, leading to type I IFN and chemokine production [23]. While type I IFN enhances dendritic cells maturation and T cells activation [24], STING signaling also promotes immunosuppression by inducing CCL2 and CCL7, which recruit monocytic myeloid‐derived suppressor cells (M‐MDSCs) [25]. Emerging evidence suggests that STING is regulated not only by post‐translational modifications but also through post‐transcriptional mechanisms [26]. For example, the RNA‐binding protein LUC7L2 promotes intron 3 retention in STING pre‐mRNA, thereby reducing STING protein expression [27]. Given the established role of RBM10 in alternative splicing, we hypothesized that RBM10 may regulate STING splicing and consequently shape the tumor immune microenvironment. As ligands of CCR2, CCL2 and CCL7 are regarded as promising anticancer therapeutic targets [28, 29]. However, current strategies have focused predominantly on STING activation, whereas approaches that concurrently target STING signaling and suppress downstream immunosuppressive chemokines remain underexplored.

We have previously demonstrated that RBM10 functions as a tumor suppressor in LUAD [30, 31]; but its role in antitumor immunity has not been defined. The present study identified a novel function for RBM10 in regulating macrophage polarization and recruitment. The findings revealed that RBM10 deficiency promotes STING exon 3 (E3) inclusion, activating the cGAS‐STING pathway and inducing CCL7 secretion, which drives macrophage‐mediated immune evasion. Notably, CCL7/CCR2 blockcade restores ICIs efficacy in RBM10‐low LUAD, suggesting a potential combination strategy. A schematic representation of study is presented in Figure S12.

## 2 Results

### 2.1 RBM10 Expression Correlates With Response to PD‐1 Inhibitor and Prognosis in LUAD Patients

**FIGURE 1:**
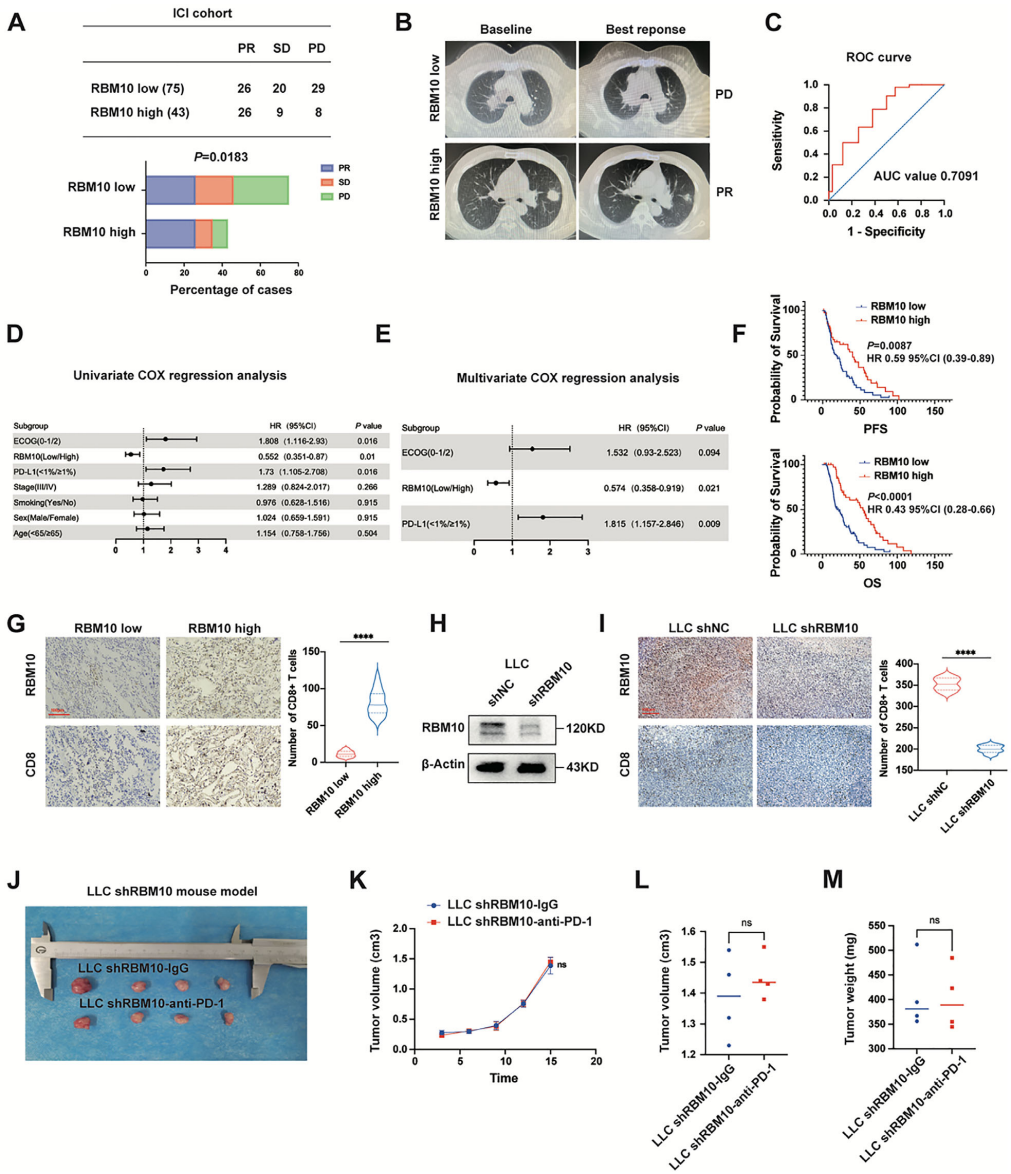
RBM10 expression associates with immunotherapy efficacy and prognosis in LUAD patients. (A) Bar chart showing the distribution of treatment responses (PR, SD, PD) among LUAD patients with high (n = 43) and low (n = 75) RBM10 expression following anti‐PD‐1 therapy. (B) Representative chest CT images of patients with high and low RBM10 expression showing PR and PD after immunotherapy. (C) ROC curve of RBM10 expression for predicting response to anti‐PD‐1 therapy (AUC = 0.7091, 95% CI: 0.618‐0.801, *P* < 0.0001). (D‐E) Forest plot of univariable and multivariable Cox regression for PFS incorporating RBM10 expression and other clinical factors. HRs with 95% CIs are shown. (F) KM survival curves comparing PFS and OS between the high and low RBM10 expression groups. (G) Representative IHC staining and quantification of CD8^+^ T‐cell infiltration in tumor tissues from LUAD patients with high versus low RBM10 expression. (H) WB validation of RBM10 knockdown in LLC cell line. (I) Representative IHC staining and quantification of CD8^+^ T‐cell infiltration in tumor tissues from mouse models. (J–M) LLC shRBM10 tumor‐bearing mice were treated with IgG or anti‐PD‐1 antibody (n  =  4). Shown are representative tumor images (J), tumor growth curves (K), and endpoint tumor volumes and weights (L,M). All data are presented as the mean ± SEM (n ≥ 3). The *P* values in panels (A) were calculated using Chi‐square test. Survival curves (F) were calculated using log‐rank test. The *P* values in panels (K) were calculated using two‐way ANOVA. The *P* values in panels (G, I and L‐M) were calculated using two‐tailed unpaired Student's *t*‐test. ns (not significant), ****P < 0.0001. Original blots can be found in File S8.

To explore the clinical relevance of RBM10 in immunotherapy, we retrospectively analyzed a cohort of 118 patients with LUAD who received PD‐1 inhibitor‐based treatment. Based on RBM10 expression levels determined by immunohistochemistry (IHC), patients were stratified into low (n = 75) and high (n = 43) expression groups. The distribution of treatment responses including partial response (PR), stable disease (SD) and progressive disease (PD) differed significantly between the two groups, with patients harboring low RBM10 expression exhibiting poorer responses to immunotherapy (Figure 1A). Representative chest CT images illustrating PR and PD in patients with high and low RBM10 expression, respectively, are shown in Figure 1B. Receiver operating characteristic (ROC) curve analysis in Figure 1C also showed that RBM10 expression predicted immunotherapy objective response with an area under the curve (AUC) of 0.7091(95% confidence interval: 0.618‐0.801, *P* < 0.0001).

Univariable and multivariable Cox regression showed that high RBM10 expression and PD‐L1<1% were independently associated with longer progression‐free survival (PFS) (hazard ratio = 0.574 and 1.815, respectively, both *P* < 0.05) (Figure 1D‐E). Consistently, Kaplan‐Meier (KM) survival analysis revealed that patients with high RBM10 expression had significantly prolonged PFS and OS compared to those with low RBM10 expression (Figure 1F). IHC analysis of the TME further demonstrated significantly increased CD8^+^ T cells infiltration in tumors with high RBM10 expression (Figure 1G).

To functionally validate the role of RBM10 in immunotherapy response, we established subcutaneous Lewis lung carcinoma (LLC) tumor models using shNC (control) and shRBM10 cells (Figure 1H). Consistent with the clinical observations, IHC analysis of these mice tumors also revealed significantly increased CD8^+^ T cells infiltration in RBM10‐high tumors (Figure 1I). To evaluate therapeutic efficacy, RBM10‐deficient tumor‐bearing mice were treated with either IgG isotype control or anti‐PD‐1 antibody. RBM10‐deficient tumors showed no significant response to PD‐1 inhibitor, with comparable tumor volumes and weights between treatment groups at the experimental endpoint (Figure 1K–M). Collectively, these findings identify RBM10 as a critical modulator of immunotherapy efficacy in LUAD, warranting further mechanistic investigation.

### 2.2 RBM10 Regulates M2 Polarization and Infiltration in the LUAD TME

To investigate the association between RBM10 expression and macrophage polarization in LUAD, we performed comprehensive immune deconvolution analysis of The Cancer Genome Atlas (TCGA)‐LUAD cohort using multiple established algorithms, including CIBERSORT, MCP‐COUNTER, TIMER, xCell, and quanTIseq. Across all analytical methods, RBM10 transcript levels showed a strongly positive correlation with M1 macrophage infiltration and a pronounced negative correlation with M2 macrophage infiltration (Figure S1A). Consistently, tumors with high RBM10 expression exhibited significantly increased M1 macrophage infiltration and decreased M2 macrophage accumulation compared with low RBM10‐expressing tumors (Figure S1B).

To validate the functional impact of RBM10 on tumor growth and macrophage polarization in vivo, we confirmed efficient RBM10 depletion in shRBM10 LLC and CMT167 tumors by western blotting (WB) analysis (Figure 1I; Figure S1C). Tumors derived from LLC shRBM10 cells exhibited significantly enhanced growth, with increased final tumor volumes and weights compared with shNC controls (Figure S1D–F). Flow cytometry (FC) analysis of tumor‐infiltrating immune cells (gated on live CD45^+^ CD11b^+^ F4/80^+^ macrophages) revealed substantial polarization shifts in both LLC and CMT167 shRBM10 tumors. Specifically, the proportion of M1‐like macrophages (CD86^+^, Q1) decreased, whereas that of M2‐like macrophages (CD206^+^, Q3) increased, relative to the shNC group (Figure S1G,H). Collectively, these polarization shifts support the bioinformatic predictions of RBM10‐mediated immunomodulation.

### 2.3 Macrophage Landscape in Murine Tumors With Differential RBM10 Expression

**FIGURE 2:**
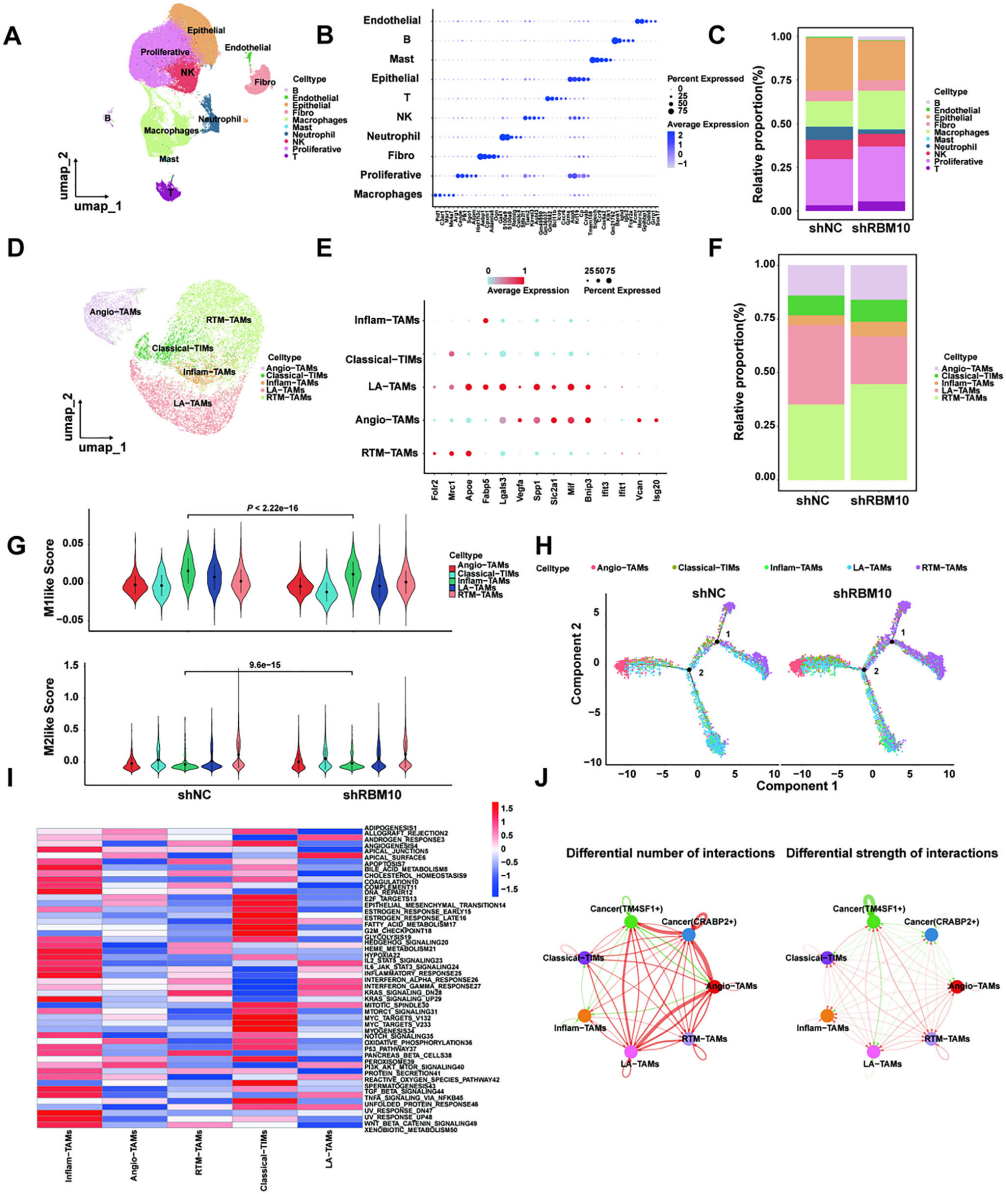
Macrophage landscape in mouse tumors with differential RBM10 expression. (A) UMAP visualization illustrating the cell types identified in LLC shNC (n = 3) and shRBM10 (n = 3) tissues. (B) Dot plot showing expression of marker genes for each cell cluster. (C) Bar chart showing proportions of distinct cell populations. (D) UMAP visualization of macrophage subpopulations. (E) Dot plot showing marker genes for each macrophage subset. (F) Bar chart showing proportions of macrophage subsets. (G) Violin plots comparing M1 and M2 polarization scores across subsets (n = 3). (H) Pseudotime trajectory analysis of macrophage differentiation. (I) Heatmap of functional pathways enriched in macrophage subsets. (J) Cell chat analysis showing the interaction strength and number between malignant epithelial cells and macrophage subsets (red: upregulated in shRBM10; green: downregulated). All data are presented as the mean ± SEM (n = 3). The *P* values in panels (G) were calculated using two‐tailed paired Student's *t*‐test.

To further characterize the impact of RBM10 on the tumor immune landscape, we performed single‐cell RNA sequencing (scRNA‐seq) on tumor tissues from LLC shNC (n = 3) and shRBM10 (n = 3) C57BL/6 mice. Cell subpopulations were annotated based on marker gene expression and visualized using Uniform Manifold Approximation and Projection (UMAP) dimensionality reduction. Clustering analysis identified 10 major cell subpopulations (Figure 2A), with their respective marker genes further characterized (Figure 2B). Comparison of cell type proportions between the two groups revealed increased macrophage infiltration in shRBM10 tumors, suggesting enhanced macrophage recruitment upon RBM10 knockdown (Figure 2C).

Based on established classification markers [32], macrophages were further categorized into 5 subpopulations: Angio‐TAMs, Classical‐TIMs, Inflam‐TAMs, LA‐TAMs, and RTM‐TAMs (Figure 2D). Representative marker genes were identified as follows: RTM‐TAMs (FOLR2, MRC1); LA‐TAMs (APOE, FABP5, LGALS3); Angio‐TAMs (VEGFA, SPP1, SLC2A, MIF, BNIP3); Inflam‐TAMs (FABP5); and Classical‐TIMs (MRC1) (Figure 2E). Following RBM10 knockdown, the overall abundance of all macrophage subsets increased significantly (Figure 2C). Notably, the proportions of RTM‐TAMs and Angio‐TAMs were preferentially elevated, whereas the proportion of LA‐TAMs decreased (Figure 2F).

To assess functional polarization, the 5 subsets were scored using M1 and M2 signature gene sets (Supplementary Table S4). Compared with the shNC group, shRBM10 tumors exhibited reduced pro‐inflammatory (M1) scores and elevated anti‐inflammatory (M2) scores, with the most pronounced shifts observed in Classical‐TIMs and LA‐TAMs (Figure 2G). Notably, although the relative proportion of LA‐TAMs decreased, the remaining LA‐TAMs displayed a more strongly polarized M2‐like phenotype, indicating enhanced immunosuppressive activity at the single‐cell level. Developmental trajectory analysis also indicated that RTM‐TAMs preferentially differentiated toward LA‐TAMs, which exhibited immunosuppressive functions, in the setting of RBM10 knockdown (Figure 2H). Functional enrichment analysis further revealed that LA‐TAMs were significantly enriched in inflammatory response‐related pathways, including inflammatory response, IL6‐JAK‐STAT3 signaling, and IL2‐STAT5 signaling (Figure 2I).

Malignancy scoring identified TM4SF1^+^ and CRABP2^+^ epithelial cells as malignant populations. Cell‐cell interaction analysis using Cell Chat revealed that RBM10 knockdown markedly increased the number of interactions between these malignant epithelial cells and macrophage subsets, particularly between TM4SF1^+^ cancer cells and LA‐TAMs, and between CRABP2^+^ cancer cells and Angio‐TAMs, whereas interaction strength was not significantly altered (Figure 2J). Together, these findings indicate that RBM10 deficiency alters the composition, polarization, and intercellular communication of macrophage subsets in the LUAD TME, supporting a pro‐tumorigenic and immunosuppressive landscape.

### 2.4 RBM10 Modulates Macrophage Recruitment and Polarization in Tumor Cell‐Macrophage co‐Culture System

To investigate the role of RBM10 in macrophage recruitment and polarization, we established a co‐culture system using LUAD cells (PC9, 3255, and 292) and macrophages (Figure S2A). Knockdown or overexpression (OE) of RBM10 in LUAD cells was validated by quantitative real‐time PCR (qPCR) and WB (Figures S2B and S3A,B). Phorbol 12‐myristate 13‐acetate (PMA)‐induced THP‐1‐derived M0 macrophages exhibited increased CD68 expression by qPCR and FC, confirming successful differentiation (Figures S2C and S3C).

Co‐culture with RBM10‐deficient LUAD cells (PC9 shRBM10, 3255 shRBM10, and 292 vector) promoted an immunosuppressive M2‐like phenotype in macrophages, as evidenced by significantly upregulated M2 markers (CD206, CD163, ARG1, IL‐10) and downregulated M1 markers (TNF‐α, CXCL10) at the transcriptional level (Figures S2D, S3D,L). Consistently, FC and immunofluorescence (IF) analyses revealed that CD206 expression was significantly increased, whereas CD86 expression was decreased, in macrophages co‐cultured with RBM10‐deficient LUAD cells compared with those co‐cultured with RBM10‐proficient controls (Figures S2E‐F, I‐J and S3E, H‐J). WB further confirmed elevated ARG1 and reduced CD86 protein levels in macrophages under RBM10‐deficient co‐culture conditions (Figures S2K and S3G, K). In addition, conditioned medium (CM) from RBM10‐low LUAD co‐culture systems enhanced macrophage chemotaxis, indicating increased chemoattractant activity (Figures S2G,H and S3F, M,N). Collectively, these findings demonstrate that RBM10 deficiency in LUAD cells facilitates macrophage recruitment and promotes M2 polarization.

### 2.5 RBM10‐Mediated M2 Macrophage Reprogramming Facilitates LUAD Malignancy

To evaluate the functional impact of RBM10‐modulated macrophages on LUAD cell behavior, CM was collected from RBM10‐modified tumor cell‐macrophage co‐culture systems and applied to tumor cells for functional assays (Figure S4A). CCK‐8 and colony formation assays revealed that CM from PC9‐shRBM10‐M co‐cultures significantly enhanced tumor cell proliferation, whereas CM from 292 OE‐M co‐cultures suppressed proliferation, compared with their respective controls (PC9 shNC‐M or 292 vector‐M) (Figure S4B‐E). To assess the effects on migration, transwell migration and wound healing assays were performed. Tumor cells exposed to CM from PC9‐shRBM10‐M co‐cultures exhibited markedly increased migratory capacity, whereas those exposed to CM from 292 OE‐M co‐cultures showed reduced migration, relative to controls (Figure S4F‐I). Parallel Matrigel invasion assays yielded results consistent with this bidirectional regulation, further supporting the RBM10‐dependent modulation of invasive potential (Figure S4H‐I).

To validate the role of macrophages in RBM10‐mediated tumorigenesis in vivo, C57BL/6 mice were treated with clodronate liposomes (5 mg/kg) or phosphate‐buffered saline (PBS) for systemic macrophage depletion. One week after depletion, LLC shNC or shRBM10 cells were subcutaneously injected into treated mice (n = 5 per group). Clodronate‐mediated macrophage depletion significantly attenuated the pro‐tumorigenic effects of RBM10 knockdown, as evidenced by reduced tumor volume and weight compared with non‐depleted controls (Figure S4J‐L). Collectively, these findings demonstrate that RBM10 deficiency enhances LUAD malignancy by promoting M2 macrophage polarization and function.

### 2.6 RBM10 Regulates Macrophage Phenotype Within TME Through STING Mediated CCL7 Secretion

**FIGURE 3:**
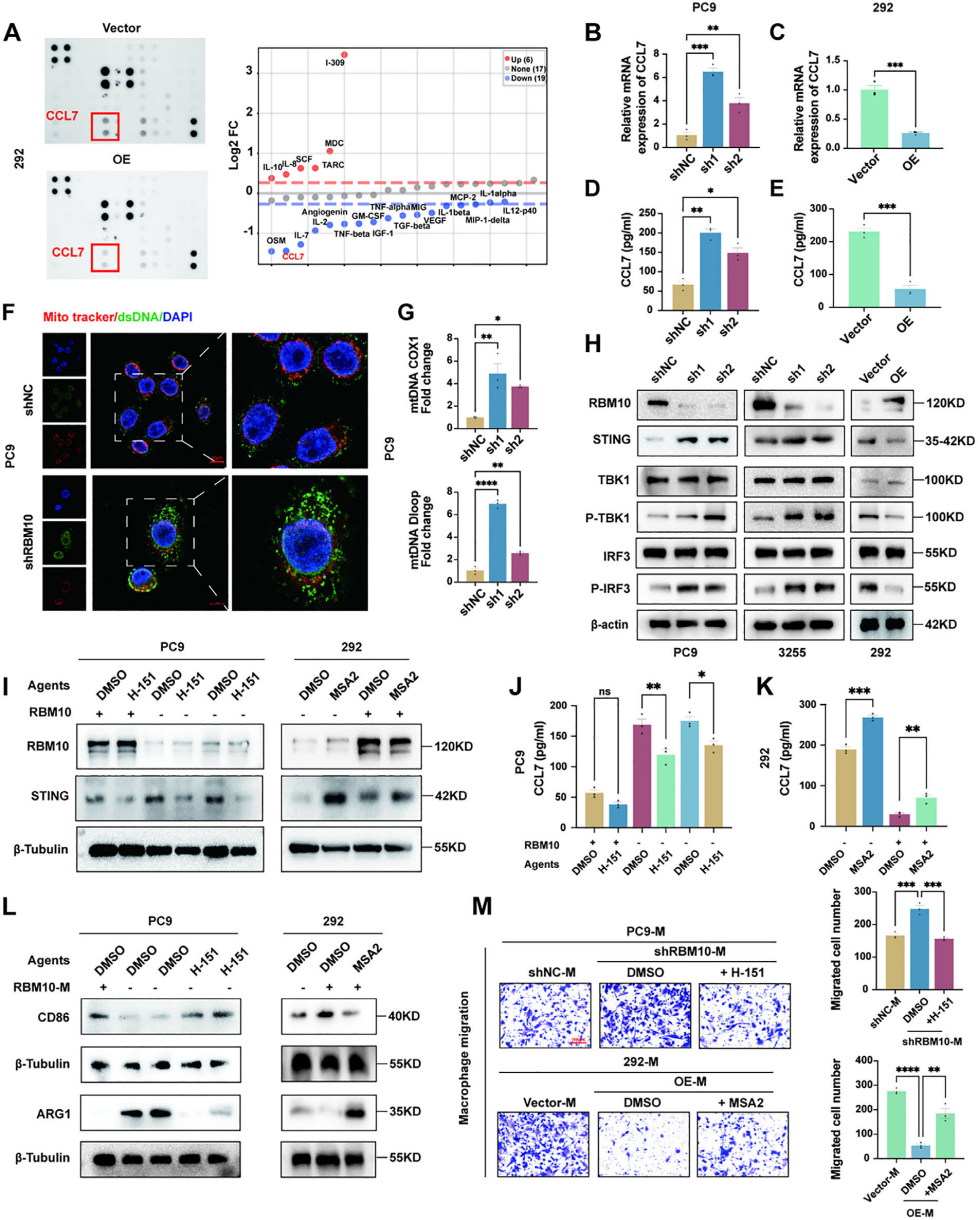
RBM10 regulates macrophage phenotype in LUAD through STING mediated CCL7 secretion. (A) Cytokine antibody array comparing 42 cytokines in 292 vector and 292OE cells (FC ≥ 1.2 or < 0.83, n = 3). (B‐C) qPCR analysis of CCL7 mRNA in PC9 and 292 cells (n = 3). (D,E) ELISA of secreted CCL7 in PC9 and 292 cells (n = 3). (F) Confocal microscopy of mtDNA (green), mitochondria (MitoTracker, red), and nuclei (DAPI, blue) in PC9 shNC and shRBM10 cells. (G) qPCR quantification of cytosolic mtDNA in PC9 shNC and shRBM10 cells (n = 3). (H) WB of cGAS‐STING pathway components in PC9 and 292 cells. (I) WB of STING in PC9 and 292 cells treated with H‐151 (1 µM, 24 h) or MSA‐2 (5 µg/mL, 24 h) versus DMSO. (J‐K) ELISA of CCL7 in PC9 and 292 cells treated with H‐151 or MSA2 (n = 3). (L) WB of CD86 and ARG1 expression in macrophages co‐cultured with treated PC9 and 292 cells. (M) Transwell assay of macrophage recruitment induced by CM from treated PC9 and 292 cells (n = 3). All data are presented as the mean ± SEM (n = 3). The *P* values in panels (B, D, G and M) were calculated using one‐way ANOVA. The *P* values in panels (C, E) were calculated using two‐tailed unpaired Student's *t*‐test. The *P* values in panels (J‐K) were calculated using two‐way ANOVA. *P < 0.05, **P < 0.01, ***P < 0.001, ****P < 0.0001. Original blots can be found in File S8.

To investigate how tumor‐intrinsic RBM10 regulates macrophage phenotypes, we analyzed CM from RBM10‐proficient and ‐deficient LUAD cells using cytokine antibody arrays targeting 42 soluble factors. Among the cytokines screened, oncostatin M (OSM), CCL7, and IL‐7 were the most markedly downregulated in CM from 292 RBM10 OE cells based on predefined criteria (fold change ≥ 1.2 or < 0.83) (Figure 3A, Table S5). Gene Ontology (GO) and Kyoto Encyclopedia of Genes and Genomes (KEGG) enrichment analyses revealed that these cytokines are involved in immune cell chemotaxis and recruitment (Figure S5A‐D). Subsequent validation by qPCR and ELISA confirmed that CCL7 mRNA and protein levels were specifically elevated in RBM10‐deficient cells, whereas OSM and IL‐7 did not exhibit consistent changes (Figure 3B‐E, Figure S5E‐H).

Previous studies have indicated that CCL7 mediates its biological functions via CCR1, CCR2, and CCR3 within TME [33, 34]. To identify the specific receptor responsible for the interaction between tumor‐derived CCL7 and macrophages, we evaluated receptor expression across four experimental groups including A: untreated M0 macrophages, B: M0 macrophages co‐cultured with PC9‐shNC cells, C: M0 macrophages co‐cultured with PC9‐shRBM10 cells, and D: M0 macrophages treated with exogenous CCL7. qPCR analysis revealed a significant upregulation of CCR2 in macrophages co‐cultured with PC9‐shRBM10 cells (C) compared to the shNC co‐culture group (B), an effect phenocopied by exogenous CCL7 treatment (D) (Figure S5I). This upregulation was further corroborated by FC analysis of surface CCR2 expression (Figure S5J). Therefore, we hypothesized that tumor‐derived CCL7 regulates macrophage phenotypes primarily through the CCR2 axis. To functionally validate this specific interaction, macrophages were treated with a CCR2‐specific blocking antibody, RS102895 or recombinant CCL7. Strikingly, CCR2 blockade abrogated the enhanced macrophage migration induced by RBM10 depletion, whereas the addition of recombinant CCL7 restored migratory capacity of macrophage in RBM10‐proficient co‐cultures (Figure S5K‐L). Collectively, these findings demonstrate that the tumor‐derived CCL7‐CCR2 signaling axis serves as a central mediator of macrophage immunosuppression driven by RBM10 deficiency.

KEGG analysis of RNA sequencing data revealed significant enrichment of the cAMP/cGMP‐PKG signaling pathway following RBM10 knockdown (Figure S6A). RBM10 deficiency also promoted mitochondrial DNA (mtDNA) release in PC9 shRBM10 cells, as evidenced by IF and qPCR analyses showing increased cytosolic mtDNA enrichment (Figure 3F‐G; Figure S6B). These findings suggested that RBM10 loss may activate the cGAS‐STING pathway, a central cytosolic DNA‐sensing mechanism involved in immune regulation and tumorigenesis [23]. Indeed, WB revealed increased phosphorylation of STING, TBK1, and IRF3 in RBM10‐deficient cells, whereas RBM10 overexpression reduced the phosphorylation levels of these signaling components (Figure 3H).

To further establish the functional link between STING activation and CCL7 secretion, we treated RBM10‐deficient cells with the STING inhibitor H‐151 and RBM10‐overexpressing cells with the STING agonist MSA2. WB confirmed that STING inhibition or activation altered STING protein expression as expected (Figure 3I). ELISA analysis showed that H‐151 reduced CCL7 secretion in PC9 shRBM10 cells, whereas MSA2 restored CCL7 secretion in 292 OE cells (Figure 3J‐K). Consistently, functional assays demonstrated that STING inhibition with H‐151 attenuated M2‐like polarization and macrophage migration in RBM10‐deficient cells, while STING activation with MSA2 promoted these effects in RBM10‐proficient cells (Figure 3L‐M). Moreover, H‐151 suppressed RBM10 deficiency‐enhanced tumor cell migration and invasion, whereas MSA2 restored the impaired invasive capacity of RBM10‐overexpressing tumor cells, as assessed by Transwell assays using CM from the co‑culture systems (Figure S6C‐F). Collectively, these results indicate that RBM10 modulates macrophage polarization and recruitment through the STING‐CCL7 axis.

### 2.7 RBM10 Regulates the AS of STING pre‐mRNA in LUAD Cells

**FIGURE 4:**
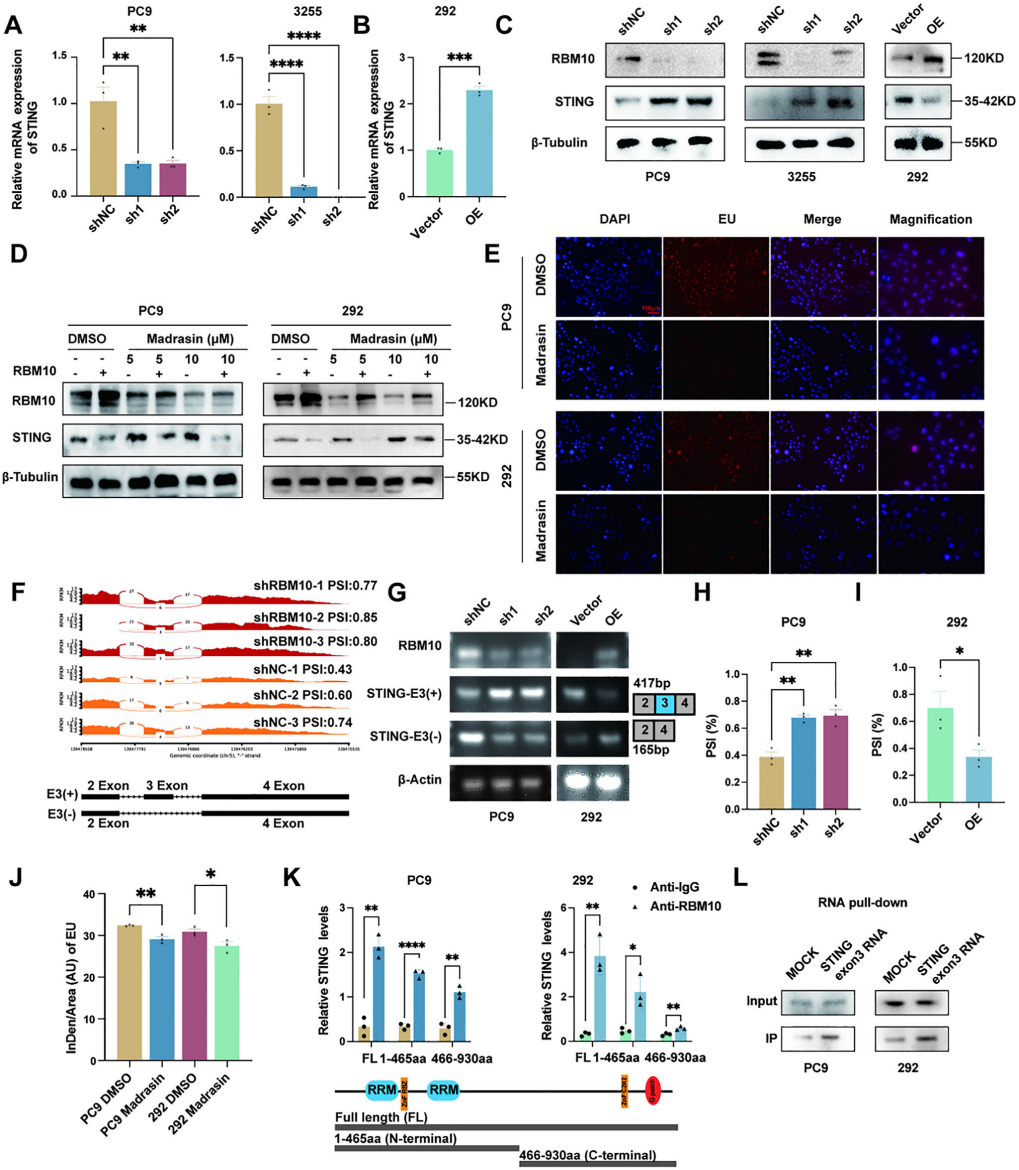
RBM10 regulates the AS of STING pre‐mRNA in LUAD cells. (A‐B) qPCR analysis of STING mRNA in PC9, 3255 and 292 cells with differential RBM10 expression (n = 3). (C) WB analysis of STING protein in indicated cells. (D) WB of RBM10 and STING in PC9 and 292 cells treated with DMSO or madrasin (5 or 10 µM, 24h). (E, J) EU assay detecting nascent RNA synthesis in PC9 and 292 cells treated with DMSO or madrasin. (F) Schematic of STING pre‐mRNA AS and PSI values from CLIP‐seq data. (G–I) RT‐PCR analysis of STING‐E3(+) and STING‐E3(‐) isoforms in PC9 and 292 cells. (K) RIP assay assessing RBM10 binding to STING pre‐mRNA in cells transfected with RBM10 domains (n = 3). (L) RNA pull‐down assay measuring RBM10 binding to STING fragments (n = 3). All data are presented as the mean ± SEM (n = 3). The *P* values in panels (A, H) were calculated using one‐way ANOVA. The *P* values in panels (B, I, J‐K) were calculated using two‐tailed unpaired Student's *t*‐test. *P < 0.05, **P < 0.01, ***P < 0.001, ****P < 0.0001. Original blots can be found in File S8.

As an RNA‐binding protein, RBM10 is known to regulate pre‐mRNA splicing [19, 20]. However, whether it modulates STING expression through AS to influence CCL7 secretion and macrophage polarization in the LUAD TME remains unclear. qPCR analysis revealed decreased STING mRNA levels in RBM10‐deficient cells, whereas WB showed elevated STING protein expression. Conversely, RBM10‐proficient cells exhibited increased STING mRNA but reduced STING protein levels (Figure 4A‐C). This inverse correlation between STING mRNA and protein levels in the context of RBM10 status suggests that RBM10 may post‐transcriptionally regulate STING expression, potentially through mechanisms involving mRNA splicing, stability, or translational efficiency.

To investigate whether RBM10 affects STING pre‐mRNA splicing, PC9 and 292 cells were treated with the splicing inhibitor madrasin (5 and 10 µM) or DMSO as a control. WB revealed that STING protein expression increased in a concentration‐dependent manner upon madrasin treatment, phenocopying the effect of RBM10 deficiency and suggesting that RBM10 loss may exert splicing‐inhibitory effects (Figure 4D). Additionally, madrasin reduced nascent RNA synthesis, as assessed by 5‐ethynyluridine (EU) labeling (Figure 4E, J), indicating that RBM10‐mediated AS contributes to STING RNA synthesis or processing.

GO and KEGG functional analyses based on cross‐linking and immunoprecipitation (CLIP)‐seq data revealed that differential RNA binding of RBM10 was predominantly associated with splicing‐related pathways (Figure S7A‐B). Among the core mechanisms of AS—exon skipping (ES), intron retention (IR), alternative 5ʹ or 3ʹ splice site selection (AA and AD), and mutually exclusive exons (MEE)—ES is the most prevalent event in mammals (>95%) [35, 36]. Nanopore long‐read sequencing of PC9 cells identified ES as the predominant RBM10‐dependent splicing event in the STING RNA (Figure S7C‐D). CLIP‐seq further identified exon 3 skipping as the major RBM10‐regulated splicing event in STING transcripts (Figure S7E). RBM10 knockdown suppressed exon 3 skipping, leading to increased levels of the STING‐E3(+) (exon 3‐inclusion) isoform and decreased levels of the STING‐E3(‐) (exon 3‐skipping) isoform, corresponding to an elevated percent spliced‐in (PSI) value [PSI = STING‐E3(+)/[STING‐E3(‐) + STING‐E3(+)] (Figure 4F). To validate these findings, reverse transcription‐polymerase chain reaction (RT‐PCR) spanning exons 2–4 was performed, generating distinct fragments corresponding to the STING E3(+) and E3(‐) isoforms. Consistent with the sequencing data, RBM10 knockdown increased the PSI value, whereas RBM10 overexpression reduced it (Figure 4G‐I). Collectively, these results indicate that RBM10 is involved in regulating STING pre‐mRNA AS.

CLIP‐seq identified major RBM10‐binding sites within STING pre‐mRNA (Figure S7F). RNA immunoprecipitation (RIP) assays using truncated RBM10 constructs showed that both the N‐terminal (1‐465 aa) and C‐terminal (466‐930 aa) fragments bind STING pre‐mRNA, with the N‐terminal region showing stronger binding (Figure 4K). RNA pull‐down assays confirmed direct binding of RBM10 to STING exon 3 (Figure 4L). Moreover, detection of RNA polymerase II CTD (YSPTSPS) phosphorylation at Ser2 on the STING promoter suggested that RBM10 may influence STING AS by modulating RNA polymerase II activity (Figure S7G‐H). Collectively, these results demonstrate that RBM10 directly binds STING pre‐mRNA at exon 3 through its N‐terminal domain, thereby regulating its AS.

### 2.8 RBM10 Modulates the Stability of STING‐E3(‐) Isoform via Quaking (QKI)

**FIGURE 5:**
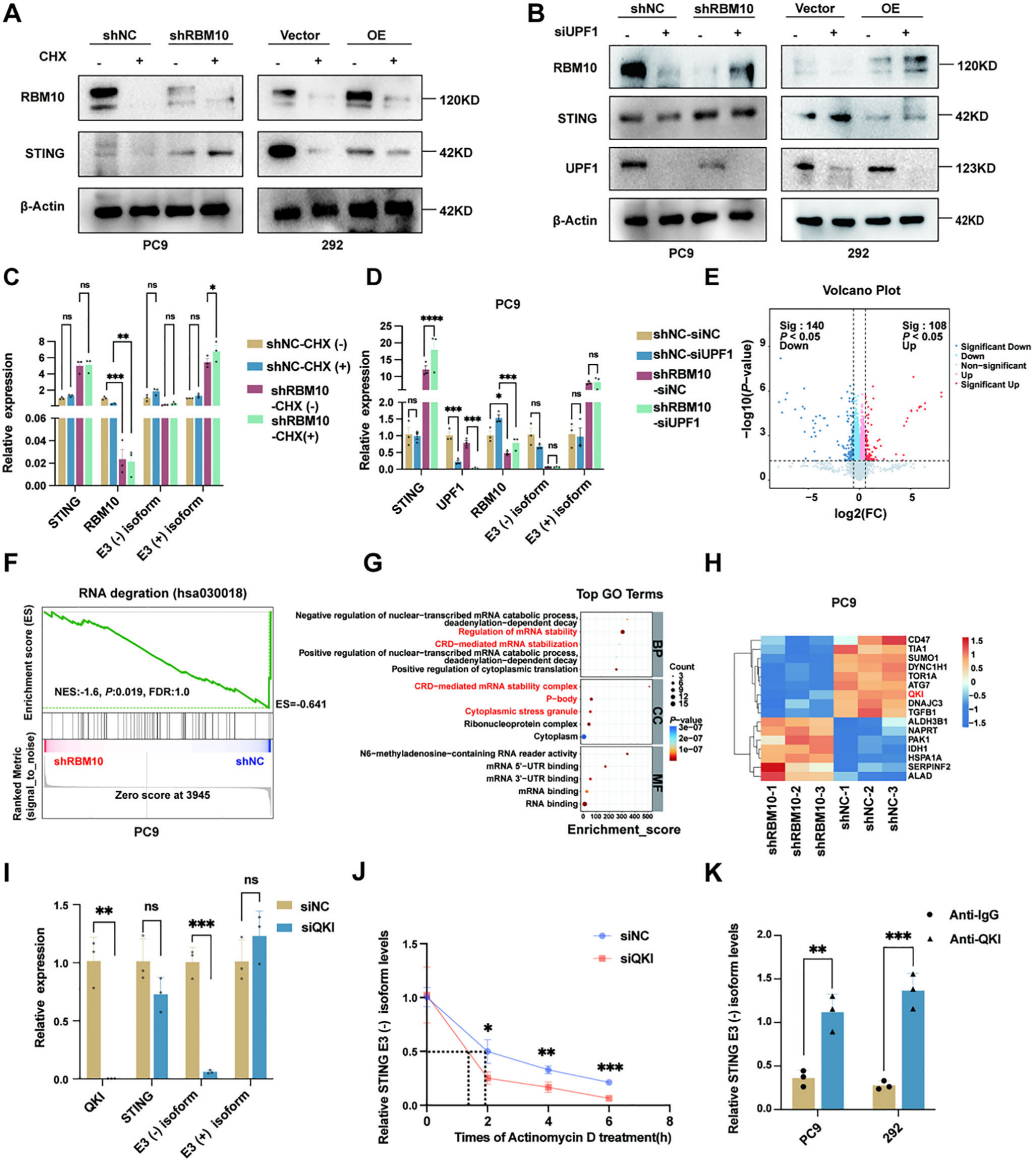
RBM10 modulates the stability of STING‐E3(‐) isoform via QKI. (A) WB of RBM10 and STING in PC9 and 292 cells treated with or without CHX (100 µg/ml, 6h). (B) WB of RBM10, STING, and UPF1 in cells with or without UPF1 knockdown. (C) qPCR analysis of indicated transcripts in cells treated with or without CHX (n = 3). (D) qPCR analysis of indicated transcripts in cells with or without UPF1 knockdown (n = 3). (E) Volcano plot of differentially expressed proteins between PC9 shNC and shRBM10 cells (n = 3). (F) GSEA showing enrichment of RNA degradation pathway. (G) GO enrichment analysis of RNA stability related proteins. (H) Heatmap displaying differentially expressed stress granule associated proteins between PC9 shNC and shRBM10 cells. (I) qPCR analysis of QKI, total STING, and STING isoforms in control and QKI‐knockdown cells (n = 3). (J) qPCR analysis of STING‐E3(‐) RNA stability after actinomycin D treatment (n = 3). (K) RIP assay assessing QKI binding to STING‐E3(‐) transcript (n = 3). All data are presented as the mean ± SEM (n = 3). The *P* values in panels (C‐D, J) were calculated using two‐way ANOVA. The *P* values in panels (I, K) were calculated using two‐tailed unpaired Student's *t*‐test. ns (not significant), *P < 0.05, **P < 0.01, ***P < 0.001, ****P < 0.0001. Original blots can be found in File S8.

Nonsense‐mediated decay (NMD) is a translation‐dependent mechanism that degrades mRNAs containing premature termination codons [37, 38]. In RBM10‐deficient cells, the STING‐E3(‐) isoform was markedly reduced, whereas the STING‐E3(+) isoform was increased. We therefore hypothesized that the STING‐E3(‐) isoform might be selectively degraded via the NMD pathway. To test this, we inhibited translation using cycloheximide (CHX) (6h, 100 µg/ml); however, CHX treatment failed to consistently restore STING‐E3(‐) expression at either the mRNA or protein level across cell lines (Figure 5A, C). Similarly, knockdown of UPF1, the central effector of NMD, failed to rescue STING‐E3(‐) expression at either the mRNA or protein level (Figure 5B, D). Collectively, these results indicate that degradation of the STING‐E3(‐) transcript occurs independently of the canonical UPF1‐mediated NMD pathway, suggesting the involvement of alternative post‐transcriptional regulatory mechanisms.

To investigate the regulatory mechanism underlying RBM10‐mediated degradation of the STING‐E3(‐) isoform, we performed comparative proteomic analysis of PC9 shNC and shRBM10 cells, which identified 248 differentially expressed proteins (DEPs), including 140 downregulated and 108 upregulated proteins upon RBM10 knockdown (Figure 5E). Gene set enrichment analysis (GSEA) revealed a significant association of these DEPs with RNA degradation and mRNA stability pathways (*P* = 0.019) (Figure 5F). Consistently, GO analysis showed enrichment in terms related to mRNA stability regulation, CRD‐mediated stabilization, P‐bodies, stress granules, and RNA binding (Figure 5G). Stress granules function as transient mRNA storage hubs during cellular stress, coordinating mRNA fate through stabilization or decay via P‐bodies to maintain RNA homeostasis [39, 40]. Heatmap profiling of stress granule‐associated proteins revealed upregulation of QKI, TIA1, SUMO1, and DYNC1H1 in PC9 shNC cells, whereas ALAD, ALDH3B1, and NAPRT were elevated in PC9 shRBM10 cells (Figure 5H). Notably, QKI was significantly downregulated upon RBM10 knockdown (fold change = 0.67, *P* < 0.05), suggesting a key role in RBM10‐mediated stabilization of spliced isoforms.

QKI is a well‐characterized RNA‐binding protein that recognizes specific RNA motifs, such as ACUAAY in 3′‐UTRs, to regulate transcript stability and influence cancer progression [39, 41, 42, 43]. In this study, QKI knockdown significantly downregulated STING‐E3(‐) expression (Figure 5I). Actinomycin D assays further revealed that QKI depletion reduced the stability of the STING‐E3(‐) transcript (Figure 5J), supporting its role in post‐transcriptional stabilization. Moreover, RIP assays confirmed direct binding of QKI to the STING‐E3(‐) transcript, with significant enrichment compared with IgG controls (Figure 5K). Collectively, these findings provide direct evidence of a physical interaction between QKI and the STING‐E3(‐) isoform, offering mechanistic insight into how QKI maintains STING‐E3(‐) expression through mRNA stabilization.

### 2.9 Differential Effects of STING Isoforms on Macrophage Phenotype

To explore the functional roles of individual STING splice isoforms, we performed isoform‐specific knockdown experiments in RBM10‐modified LUAD cells. Depletion of the STING‐E3(+) transcript in PC9 and 3255 shRBM10 cells reduced total STING protein levels, whereas knockdown of the STING‐E3(‐) isoform in 292 RBM10 OE cells had no significant effect on STING expression (Figure S8A‐B). These differential protein expression patterns correlated with distinct functional outcomes. ELISA analysis revealed that STING‐E3(+) knockdown significantly decreased CCL7 secretion, whereas STING‐E3(‐) knockdown resulted in a minor increase (Figure S8C‐D). Consistent with these secretory changes, CM from STING‐E3(+)‐deficient cells induced an M1‐like phenotype in macrophages (elevated CD86, reduced ARG1), whereas CM from STING‐E3(‐)‐deficient cells had opposed effect (Figure S8E‐F). Functionally, STING‐E3(+) depletion impaired macrophage chemotaxis, while STING‐E3(‐) deficiency enhanced it (Figure S8G–J).

To assess the functional sufficiency of individual STING splice isoforms, we overexpressed STING‐E3(+) and STING‐E3(‐) in RBM10‐proficient PC9 and 3255 cells. Overexpression of STING‐E3(+), but not STING‐E3(‐), significantly increased total STING protein levels (Figure S9A) and markedly enhanced CCL7 secretion (Figure S9C). Functionally, CM from STING‐E3(+) overexpressing cells promoted an M2‐like macrophage phenotype, characterized by increased ARG1 expression and reduced CD86 levels, and significantly enhanced macrophage chemotaxis (Figure S9E‐G). In contrast, STING‐E3(‐) overexpression exerted a weak but opposed effect on macrophage polarization and migration compared with STING‐E3(+) (Figure S9B, D, E, H‐I). Collectively, these data establish that the STING‐E3(+) isoform is both necessary and sufficient to drive CCL7 secretion and M2 macrophage polarization downstream of RBM10 deficiency, acting as the primary regulator of STING protein expression and downstream functional activity in macrophages.

### 2.10 Mitochondrial Transfer Reinforces the STING‐CCL7 Axis as a Positive Feedback Loop

**FIGURE 6:**
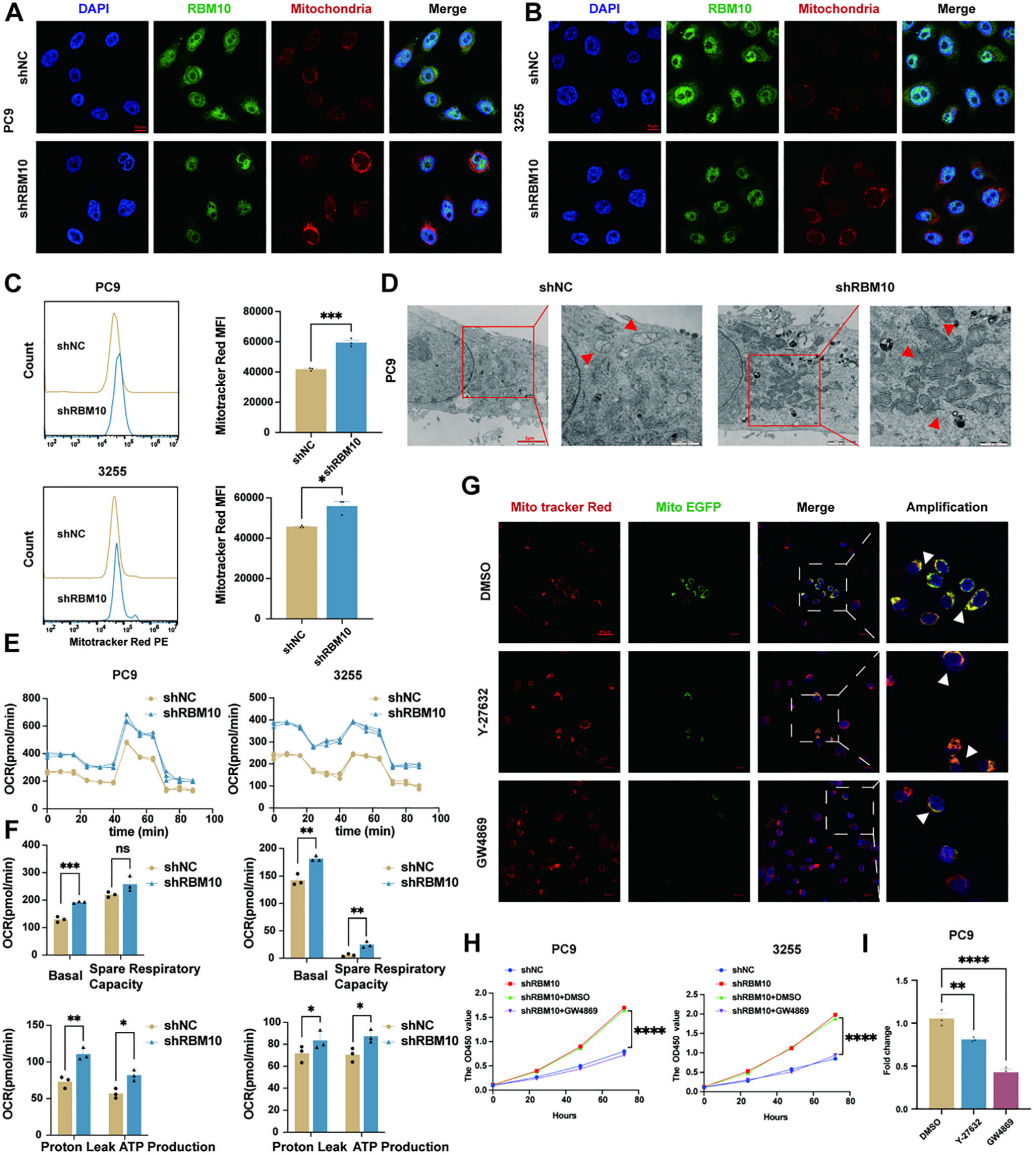
Mitochondrial transfer reinforces the STING‐CCL7 axis as a positive feedback loop. (A, B) IF of RBM10 expression and MitoTracker‐labeled mitochondrial transfer in PC9 and 3255 cells co‐cultured with M2 macrophages. (C) FC quantification of MFI in MitoTracker‐labeled mitochondria transferred to co‐cultured PC9 and 3255 cells (n = 3). (D) Electron microscopy showing mitochondrial ultrastructure in PC9 cells after co‐culture. (E, F) OCR measurements comparing basal OCR, spare respiratory capacity, proton leak and ATP production in PC9 and 3255 cells under co‐culture conditions (n = 3). (G, I) IF analysis of EGFP‐labeled mitochondrial transfer from macrophages to PC9 cells (MitoTracker Red). During co‐culture, cells were treated with DMSO, GW4869 (20 µM, 24 h), or Y‐27632 (10 µM, 24 h). (H) CCK‐8 assay of PC9 and 3255 shRBM10 cell proliferation under co‐culture with GW4869 treatment (n = 3). All data are presented as the mean ± SEM (n = 3). The *P* values in panels (C, F) were calculated using two‐tailed unpaired Student's *t*‐test. The *P* values in panels (H) were calculated using two‐way ANOVA. The *P* values in panels (I) were calculated using one‐way ANOVA. ns (not significant), *P < 0.05, **P < 0.01, ***P < 0.001, ****P < 0.0001.

Given the increased mtDNA levels in RBM10‐deficient cells and its role in cGAS‐STING activation, we hypothesized that M2‐polarized macrophages induced by RBM10 knockdown may transfer mitochondria to tumor cells, potentially contributing to sustained cGAS‐STING‐CCL7 signaling. To test this, PC9 and 3255 shNC and shRBM10 cells were co‐cultured with MitoTracker‐labeled M2 macrophages for 48h. IF and FC revealed higher mitochondrial fluorescence and mean fluorescence intensity (MFI) in shRBM10 cells compared with shNC controls, indicating enhanced mitochondrial transfer from M2 macrophages (Figure 6A–C). Ultrastructural analysis confirmed intact mitochondrial architecture (cristae and double membranes) and increased mitochondrial content in PC9 shRBM10 cells after co‐culture (Figure 6D). To assess mitochondrial function, we measured the oxygen consumption rate (OCR) in co‐cultured cells. PC9 and 3255 shRBM10 cells exhibited significantly elevated basal OCR, spare respiratory capacity, proton leak, and ATP production compared with controls (Figure 6E‐F).

Cancer cells can acquire mitochondria from neighboring cells through various mechanisms, including tunneling nanotubes, extracellular vesicles (EVs), gap junctions, and cell fusion [44]. Given the use of a tumor cell‐macrophage co‐culture system, we hypothesized that mitochondria was transferred from macrophages to tumor cells via EVs. To test this, we treated co‐cultures with DMSO, GW4869 (an inhibitor of EVs ∼200 nm), or Y‐27632 (an inhibitor of larger EVs >200 nm). IF analysis of tumor cells (MitoTracker Red) revealed that GW4869, but not Y‐27632, markedly reduced the transfer of EGFP‐labeled mitochondria from macrophages to tumor cells (Figure 6G, I). Consistently, CCK‐8 assays showed that GW4869 treatment significantly suppressed the proliferation of PC9 and 3255 shRBM10 cells under co‐culture conditions, suggesting that macrophage‐derived EVs may contribute to mitochondrial transfer and tumor cell proliferation (Figure 6H).

To determine whether mitochondrial transfer of RBM10‐deficient cells operates downstream of the core STING‐CCL7 axis, we treated PC9 and 3255 shRBM10 cells with DMSO (control), the CCR2 antagonist RS102895 and STING inhibitor H‐151 during co‐culture with MitoTracker‐labeled M2 macrophages. IF and FC analyses revealed that CCR2 and STING inhibition significantly reduced mitochondrial transfer from macrophages to tumor cells, as evidenced by decreased MitoTracker MFI in tumor cells compared with controls (Figure S10A‐C). OCR measurements further demonstrated that CCR2 and STING inhibition abrogated the enhanced basal respiration, spare respiratory capacity, and ATP production in RBM10‐deficient tumor cells induced by co‐culture with M2 macrophages (Figure S10D‐G). Collectively, these findings suggest that mitochondrial transfer in RBM10‐deficient cells may act downstream of the STING‐CCL7 axis, potentially reinforcing a positive feedback loop that promotes sustained CCL7 production and mitochondrial function.

### 2.11 CCL7‐CCR2 Axis Inhibition Potentiates Anti‐PD‐1 Therapy Efficacy in RBM10‐low LUAD

We next investigated whether macrophage‐dependent PD‐L1 regulation contributes to RBM10‐mediated immune modulation. Under monoculture conditions, PD‐L1 expression was comparable between RBM10‐proficient and RBM10‐deficient LUAD cells across multiple assays (qPCR, WB, and FC; Figure S11A‐G). In contrast, upon co‐culture with macrophages, RBM10‐deficient tumor cells exhibited significantly higher PD‐L1 expression than their RBM10‐proficient counterparts (Figure S11A‐G). This macrophage‐dependent PD‐L1 upregulation was corroborated in vivo, where RBM10‐low subcutaneous tumors showed elevated PD‐L1 expression and reduced CD8^+^ T cell infiltration compared with RBM10‐high tumors (Figure S11H‐I). Collectively, these findings indicate that PD‐L1 upregulation in RBM10‐deficient tumors occurs downstream of the STING‐CCL7‐macrophage axis, rather than as a direct consequence of RBM10 loss.

**FIGURE 7:**
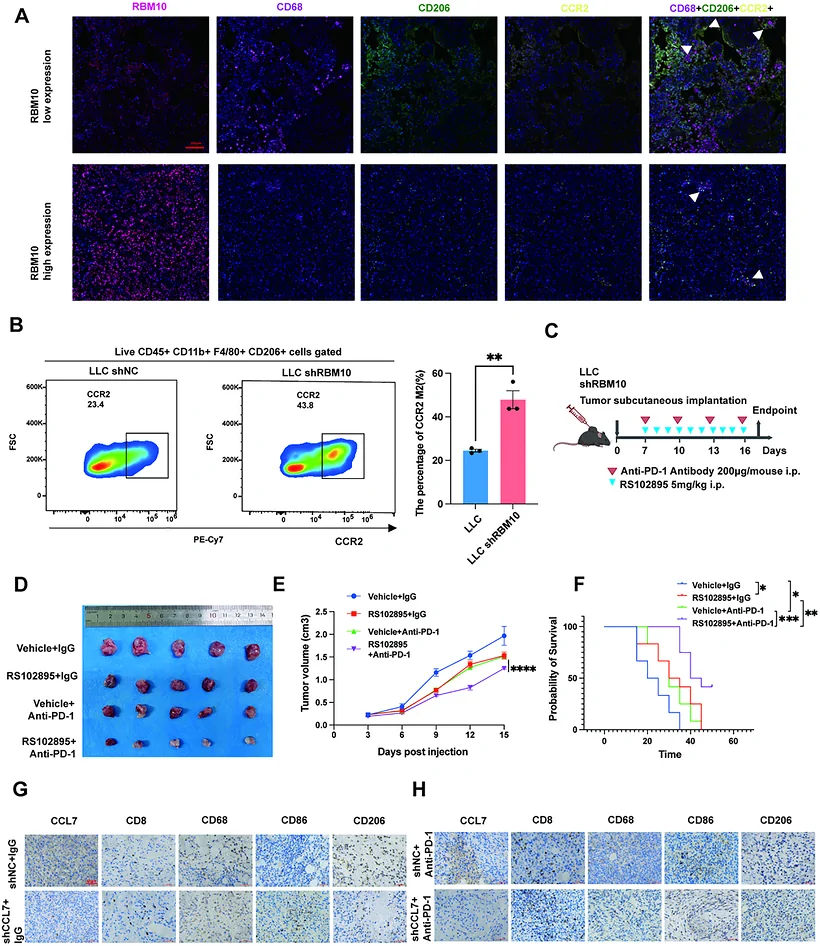
CCL7‐CCR2 axis inhibition potentiates anti‐PD‐1 therapy efficacy in RBM10‐low LUAD. (A) mIHC analysis of CCR2 expression on macrophages in tumor tissues from LUAD patients with high and low RBM10 expression. (B) FC analysis of CCR2 expression on macrophages (live^+^CD45^+^CD11b^+^F4/80^+^CD206^+^) in tumor tissues from mice with high and low RBM10 expression (n = 3). (C) Treatment schema for LLC shRBM10 tumor‐bearing mice treated with anti‐PD‐1 antibody, RS102895 or not (n = 5). [Created in BioRender. Gao, W. (2026) https://BioRender.com/awmu085] (D) Subcutaneous tumor formation in C57BL/6 injected with shRBM10 LLC cells treated with anti‐PD‐1 antibody, RS102895 or not (n = 5). (E) Tumor growth curves of indicated groups. (F) Survival curves of indicated groups. (G,H) IHC staining of CCL7, CD8, CD68, CD86, and CD206 in tumor tissues from each group. All data are presented as the mean ± SEM (n ≥ 3). The *P* values in panels (B) were calculated using two‐tailed unpaired Student's *t*‐test. The *P* values in panels (E) were calculated using two‐way ANOVA. Survival curves (F) were calculated using log‐rank test. ns (not significant), *P < 0.05, **P < 0.01, ***P < 0.001, ****P < 0.0001.

To assess the clinical relevance of CCR2 expression in macrophages, we performed multiplex immunohistochemistry (mIHC) on tumor tissues from LUAD patients. The proportion of CD68^+^CD206^+^CCR2^+^ cells was significantly elevated in patients with low RBM10 expression compared with those with high RBM10 expression (Figure 7A). Consistently, FC analysis of murine tumor tissues revealed a marked increase of CCR2 expression in live CD45^+^CD11b^+^F4/80^+^CD206^+^ cells in RBM10‐low tumors relative to RBM10‐high controls (Figure 7B). To evaluate whether blocking the immunosuppressive tumor‐macrophage crosstalk via CCL7 inhibition enhances anti‐PD‐1 efficacy, we established subcutaneous tumor models using RBM10‐low LUAD cells (LLC shRBM10). Starting on day 7, mice were treated with anti‐PD‐1 or IgG control, with or without CCL7 knockdown or the CCR2 antagonist RS102895 (Figure 7C; Figure S11J). Both CCL7 knockdown and RS102895 treatment significantly suppressed tumor growth and enhanced the anti‐tumor effect of anti‐PD‐1 therapy compared with controls (Figure 7D–F; Figure S11K‐M). IHC analysis of tumors from CCL7‐knockdown mice revealed increased CD8^+^ T cell infiltration and reduced M2‐like macrophage accumulation (Figure 7G‐H). These findings suggest that CCL7 inhibition converts RBM10‐low LUAD into immunologically “hot” tumors, thereby enhancing responsiveness to ICIs.

### 2.12 RBM10 Expression Combined With Macrophage Markers Refines Prognostic Stratification in LUAD Patients Receiving Immunotherapy

**FIGURE 8:**
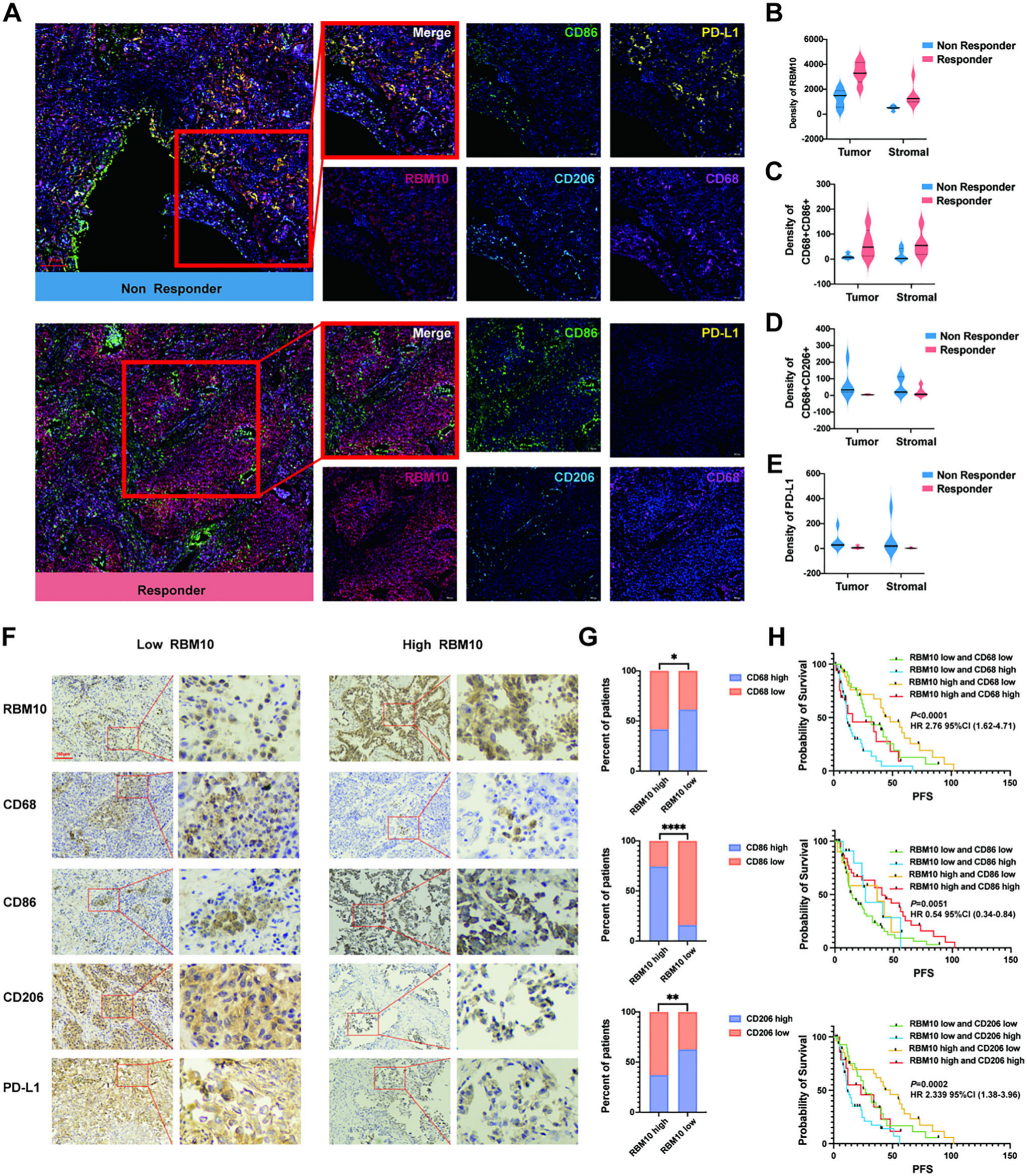
RBM10 expression combined with macrophage markers refines prognostic stratification in LUAD patients receiving immunotherapy. (A) mIHC showing the infiltration of RBM10^+^, CD68^+^, CD86^+^, CD206^+^, and PD‐L1^+^ cells in the TME of LUAD responder (n = 4) and non‐responder (n = 4) to immunotherapy. (B‐E) Violin plots displaying the density of RBM10^+^ cells, CD68^+^CD86^+^ cells, CD68^+^CD206^+^ cells, and PD‐L1^+^ cells in the tumor and stromal regions of responder (n = 4) and non‐responder (n = 4) to immunotherapy. (F) Representative IHC images of RBM10, CD68, CD86, CD206, and PD‐L1 expression in RBM10‐high (n = 43) and RBM10‐low (n = 75) groups. (G) Bar charts quantifying the proportions of CD68^+^, CD86^+^, and CD206^+^ cells in RBM10‐high (n = 43) and RBM10‐low (n = 75) groups. (H) KM survival curves illustrate PFS differences in patients stratified by RBM10 expression in combination with macrophage markers (CD68, CD86, CD206) (n = 118). All data are presented as the mean ± SEM (n ≥ 3). The *P* values in panels (G) were calculated using Chi‐square test. Survival curves (H) were calculated using log‐rank test. *P < 0.05, **P < 0.01, ****P < 0.0001.

To explore the cellular basis underlying the association between RBM10 expression and immunotherapy response, we performed mIHC on tumor biopsies from a subset of 8 patients within the 118‐patient immunotherapy cohort. This analysis enabled simultaneous quantification of RBM10, CD68 (pan‐macrophage), CD86 (M1‐like), CD206 (M2‐like), and PD‐L1 expression (Figure 8A). Quantitative fluorescence analysis revealed that, compared with responders, non‐responders exhibited significantly lower RBM10 expression and fewer CD68^+^CD86^+^ cells, but higher CD68^+^CD206^+^ cell infiltration and PD‐L1 expression (Figure 8B‐E).

Extending these observations to the full immunotherapy cohort (n = 118), we stratified patients based on RBM10 expression and quantified macrophage infiltration (CD68^+^, CD86^+^, and CD206^+^) by IHC (Figure 8F‐G). Consistent with the mIHC findings, RBM10‐low tumors exhibited significantly higher CD68^+^ macrophage infiltration, M2‐like (CD206^+^) polarization, and PD‐L1 expression, along with reduced M1‐like (CD86^+^) macrophages compared with RBM10‐high tumors (Figure 8F). Correlation analyses between RBM10 and macrophage marker expression levels were consistent with prior findings (Table S6).

To assess whether combining RBM10 expression with macrophage markers enhances prognostic value, we performed survival analyses in the 118‐patient cohort. Patients with RBM10‐high/CD68‐low or RBM10‐high/CD206‐low TME exhibited superior outcomes, whereas the RBM10‐low/CD86‐low subgroup had the poorest prognosis (Figure 8H). Collectively, these clinical findings support the mechanistic model in which RBM10 deficiency promotes tumor progression by enhancing TAMs infiltration and pro‐tumor polarization, and suggest that incorporating macrophage markers may refine patient stratification for immunotherapy.

## 3 Discussion

Although RBM10 is well established as a key regulator of cancer cell proliferation, apoptosis, and metastasis [19, 30, 31, 45], its immunomodulatory functions within the TME remain largely unexplored. By integrating bulk and scRNA seq analyses, our study demonstrates that RBM10 significantly influences responsiveness to ICIs by remodeling the TME in LUAD, particularly through promoting the infiltration of immunosuppressive macrophages. Mechanistically, RBM10 deficiency promotes a CCL7‐mediated macrophage polarization axis that may be reinforced by a positive feedback loop involving mitochondrial transfer. Collectively, these findings indicate that RBM10 plays a pivotal role in antitumor immunity in LUAD and provide a molecular framework for exploring the roles of RBM family members in cancer immunobiology across diverse tumor types.

The bidirectional crosstalk between tumor cells and TAMs has been extensively investigated, with tumor‐derived factors modulating M1/M2 polarization and TAMs reciprocally influencing tumor progression through paracrine cytokine signaling [46, 47, 48]. For instance, integrin β8 (ITGβ8) promotes LUAD progression by activating the PI3K/AKT/IRF9/CCL5 pathway to recruit and polarize macrophages; in turn, TAM‐derived IL‐8 and IL‐10 upregulate ITGβ8 in tumor cells via SPI1 [13]. Similarly, in cholangiocarcinoma, tumor cells induce M2‐like TAMs that secrete IL‐10, accelerating tumor progression through STAT3‐mediated growth, motility, and epithelial‐mesenchymal transition (EMT) [49]. In our study, RBM10 deficiency drives STING alternative splicing to establish a CCL7‐mediated macrophage polarization axis as a central mechanism. This axis may be further reinforced by a positive feedback loop in which M2‐polarized TAMs transfer mitochondria to tumor cells, potentially sustaining CCL7 production through mtDNA‐cGAS‐STING signaling. However, although mitochondrial transfer correlated with enhanced STING activation, its mechanistic contribution to mtDNA‐cGAS‐STING signaling remains to be defined.

The cGAS‐STING signaling pathway functions as a critical innate immune surveillance mechanism that regulates multiple stages of antitumor immunity. This cytosolic DNA‐sensing pathway induces type I IFN production and immune activation through recruitment of effector cells such as T and NK cells [22, 50, 51]. However, STING activation can also exert immunosuppressive effects by stimulating the release of inhibitory cytokines and chemokines within the TME [52], creating a paradoxical outcome that may facilitate recruitment of immunosuppressive cell populations. For instance, STING‐dependent NF‐κB signaling promotes IL‐6 secretion and STAT3 phosphorylation, driving tumor cell survival, PD‐L1 upregulation, and immunosuppression [53]. Moreover, radiation‐induced STING activation upregulates CCL2 and CCL7, promoting M‐MDSCs recruitment and immunosuppression in colon cancer [54], while STING activation by a gemcitabine‐based polymer similarly upregulates CCL2 and CCL7 to recruit TAMs and M‐MDSCs in pancreatic cancer [25].

RBM10 is an RNA‐binding protein that regulates AS during tumor development, progression, and therapeutic response [18]. In EGFR‐mutant LUAD, RBM10 deficiency upregulates the anti‐apoptotic Bcl‐xL isoform, impairing sensitivity to osimertinib [55]. In HCC, RBM10 loss impairs NUMB exon 9 skipping, activating Notch signaling and CCL2 expression, which drives macrophage polarization and immunosuppression [21]. Building on these findings, our study is the first to report that RBM10 regulates macrophage phenotype through splicing‐dependent mechanisms in LUAD, thereby reshaping immunotherapy responses.

Our findings suggest that RBM10 deficiency promotes STING‐E3(+) dominance through altered alternative splicing and impaired QKI‐mediated stabilization of the STING‐E3(‐) transcript, thereby enhancing CCL7 secretion and downstream immunosuppressive effects. QKI is known to stabilize target mRNAs through RNA binding and stress granule‐associated RNA preservation [39, 56, 57], consistent with our findings that QKI directly binds to the STING‐E3(‐) transcript and prolongs its half‐life (Figure 5J‐K). Given the established role of QKI in coordinating RNA fate, it will be of interest to further determine whether additional mechanisms contribute to STING‐E3(‐) stabilization. Notably, STING‐E3(‐) depletion modestly enhanced, whereas its overexpression weakly restrained CCL7 secretion and macrophage immunosuppressive phenotypes. Although the precise mechanism remains unclear, these observations may reflect altered functional balance between STING isoforms, thereby modulating STING‐E3(+)‐dependent signaling. Given the relatively modest magnitude of these effects, the regulatory role of STING‐E3(‐) warrants further investigation.

Our co‐culture experiments revealed that PD‐L1 upregulation in RBM10‐deficient LUAD cells occurs exclusively in the presence of macrophages, indicating that this effect is mediated by macrophage‐derived signals rather than intrinsic RBM10 loss. While the precise molecular mechanisms remain to be fully elucidated, emerging evidence across multiple tumor types has identified diverse macrophage‐derived signals that regulate PD‐L1 expression on tumor cells. In lung cancer, tobacco carcinogens NNK and BaP induce metabolic reprogramming of TAMs, leading to paracrine IGF2 signaling that activates insulin receptor (IR) in tumor cells and subsequently upregulates PD‐L1 via IR/NPM1‐mediated transcriptional activation [58]. In triple‐negative breast cancer, macrophage‐derived IL1β activates IL1R2 signaling in both macrophages and tumor cells, promoting PD‐L1 upregulation through the YY1/c‐Fos axis; notably, IL1R2 blockade synergized with anti‐PD‐1 therapy to suppress tumor progression [59]. In colorectal cancer, macrophage‐secreted CCL5 promotes immune escape by stabilizing PD‐L1 through the p65/STAT3‐CSN5 deubiquitination axis [60]. Collectively, these studies reveal that macrophages induce PD‐L1 expression in tumor cells through transcriptional regulation and post‐translational modification, providing candidate mechanistic frameworks for future investigation.

Anti‐PD‐1/PD‐L1 immunotherapy has revolutionized oncology by providing durable responses in previously untreatable malignancies [39, 61, 62]. Nevertheless, intrinsic and acquired resistance to ICIs remain significant clinical challenges [63]. In our study, mIHC analysis of the immunotherapy cohort identified RBM10 as a potential biomarker for PD‐1 inhibitor response in LUAD. In vivo, both CCL7 knockdown and CCR2 blockade synergized with anti‐PD‐1 therapy to suppress tumor growth in RBM10‐deficient models. Notably, accumulating evidence supports the therapeutic potential of targeting tumor cell‐intrinsic pathways that regulate macrophage function to enhance immunotherapy efficacy. Combined inhibition of the CCL2/CCR2 axis has been shown to synergize with ICIs in HCC by remodeling TAM infiltration and polarization [21]. Similarly, tumor‐specific PGAM5 deficiency enhances anti‐PD‐1 efficacy through a CCL2‐dependent mechanism, an effect that was abrogated by macrophage depletion [64]. Collectively, these findings, together with our demonstration that disrupting the CCL7‐CCR2 axis potentiates anti‐PD‐1 therapy in RBM10‐low LUAD [25, 33, 65], highlight that targeting tumor‐macrophage crosstalk represents a promising and broadly applicable strategy to overcome immunotherapy resistance.

This study has several limitations. First, the mechanisms by which macrophages induce PD‐L1 expression in RBM10‐deficient LUAD cells remain incompletely defined, and additional regulatory pathways regulating CCL7 secretion warrant further investigation. Second, while our findings establish RBM10 as a key regulator of macrophage dynamics, the TME comprises diverse immunomodulatory cell populations, including regulatory T cells and MDSCs, which may also influence therapeutic outcomes. Future studies should systematically evaluate the broader immunoregulatory functions of RBM10 across these cell types.

In summary, this study identifies a previously unrecognized axis whereby RBM10 deficiency in tumor cells drives STING‐dependent CCL7‐mediated macrophage polarization and immunosuppression in LUAD. RBM10 may serve as a potential predictive biomarker for immunotherapy response, and targeting CCL7 represents a promising strategy to overcome ICIs resistance in RBM10‐low LUAD patients.

## 4 Materials and Methods

### 4.1 Public Data Analysis and Clinical Tissue Samples

The association between RBM10 expression and macrophage infiltration was assessed using multiple immune deconvolution algorithms including MCP‐counter, CIBERSORT, and xCell within the TCGA‐LUAD cohort. For clinical validation, tumor specimens were collected from 118 patients with advanced LUAD who received anti‐PD‐1 immunotherapy at Harbin Medical University Cancer Hospital. Treatment response was assessed according to RECIST v1.1 criteria, with responders defined as patients achieving PR, and non‐responders defined as those with SD or PD. PFS and OS were used as clinical endpoints. The study protocol was approved by the Institutional Review Board of the hospital (Approval No. KY2023‐68), and written informed consent was obtained from all participants.

### 4.2 Cell Culture

PC9, H3255, and H292 (human LUAD cell lines) and THP‐1 monocytic cells, LLC and CMT167 cells were all acquired from American Type Culture Collection (ATCC). All culture medium contained 10% fetal bovine serum (FBS) (PAN Biotech GmbH, Germany) and 1% penicillin/streptomycin as supplements. The H3255, H292, THP1, LLC and CMT167 cell lines were maintained in RPMI‐1640 medium (Gibco), whereas PC9 cells were grown in DMEM (Gibco, Grand Island, NY, USA) at 37°C with 5% CO_2_ in a humidified incubator. Drug treatment conditions are described in the corresponding figure legends.

### 4.3 Single‐Cell RNA Sequencing

The scRNA seq libraries were prepared using the DNBelab C4 Single‐Cell Library Prep Set (MGI, Shenzhen) following established protocols [66]. Briefly, single‐cell suspensions were co‐encapsulated with barcoded beads in droplets, after which cells were lysed and mRNA was captured. Subsequent procedures involved breaking the emulsion, recovering the beads, and performing reverse transcription. The resulting libraries were quantified with the Qubit ssDNA Assay Kit (Thermo Fisher Scientific, USA) and sequenced on the BGI‐T1 platform at the China National GeneBank (CNGB).

### 4.4 Cell Transfection

Specific knockdown of RBM10 and mouse CCL7 was achieved using lentivirus‐delivered short hairpin RNAs (shRNAs) (GeneChem, Shanghai). A non‐targeting shRNA served as control. Viral transduction was enhanced with HitransG P (REVG005, GeneChem), and stably transduced cells were selected with 1 µg/mL puromycin for one week. Recombinant plasmids for QKI and STING isoforms were purchased from MiaoLing (Wuhan, China), and transient transfections were performed using jetPRIMER reagent (Polyplus, Cat: 101000046). shRNA/siRNA sequences are listed in Table S1. For RBM10 overexpression, lentiviral particles were generated using the Ubi‐RBM10‐3FLAG‐SV40‐puromycin vector (GeneChem) and used for cell transduction.

### 4.5 Flow Cytometry

Single‐cell suspensions were prepared from cultured cells by trypsinization, or from murine solid tumors by mechanical mincing and enzymatic digestion with collagenase (1‐2h at 37°C), followed by filtration through a 70 µm mesh. Cells were stained with Fixable Viability Dye EF506 (Thermo Fisher), then fixed and permeabilized using commercial buffers (BD Biosciences). After three washes with PBS, cells were incubated with fluorochrome‐conjugated antibodies for 30 min at 4°C in the dark. Samples were analyzed on a BD FACSAria flow cytometer, and data were processed with FlowJo software. Antibody details are listed in Table S2.

### 4.6 RNA Extraction and qPCR Analysis

Total RNA was extracted using TRIzol reagent (Invitrogen) and reverse‐transcribed to cDNA with the PrimeScript RT kit (Takara). qPCR was carried out on an ABI 7500 system (Applied Biosystems) using SYBR Green Master Mix. Gene expression was normalized to h‐actin and analyzed via the ∆∆Ct method. Primer sequences are provided in Table S3.

### 4.7 Animal Studies

Tumor‐bearing mouse models were established by subcutaneously injecting 5 × 10^6^ shNC‐ or shRBM10‐transduced LLC or CMT167 cells into the axilla of 4‐ to 6‐week‐old female C57BL/6 mice (Harbin Medical University). The cells were suspended in 150 µL of a 1:1 mixture of PBS and Matrigel. All animal procedures were approved by the Institutional Animal Care Committee. Mice were sacrificed 35 days post‐inoculation for tumor collection. Tumor volume was calculated as (width^2^ × length)/2, and tumor weight was recorded prior to FC or IHC analysis.

To deplete macrophages, clodronate‐loaded liposomes (FormuMax Scientific Inc.) were administered intravenously. An initial dose of 200 µL was given 48h before LLC cell implantation, followed by 100µL every 5 days to maintain depletion (n = 5 per group) (FigureS4J‐L). For anti‐PD‐1 monotherapy (Figure 1), mice bearing LLC shRBM10 tumors were treated with anti‐PD‐1 antibody (200 µg/mouse, MCE, HY‐P990824) or IgG control via intraperitoneal injection twice weekly for two weeks. To evaluate combination strategies (Figure 7C), one week after subcutaneous injection of shRBM10 LLC cells, mice were randomly divided into four groups (n = 5 each): shNC + IgG, shCCL7 + IgG, shNC + anti‐PD‐1, and shCCL7 + anti‐PD‐1, receiving the same anti‐PD‐1 or IgG regimen. In a separate cohort for CCR2 antagonist combination (Figure S11K), shRBM10 tumor‐bearing mice received daily intraperitoneal injections of RS102895 (5 mg/kg, MCE, HY‐18611) together with anti‐PD‐1 (twice weekly) or vehicle controls for 10 consecutive days. The study protocol was approved by the Institutional Review Board of the hospital (Approval No. KY2023‐68).

### 4.8 Statistical Analysis

Data were analyzed using GraphPad Prism 8.0 and R 4.3.1. Quantitative data are presented as mean ± SEM with a sample size of n ≥ 3 for all experiments. Comparisons between two groups used two‐tailed unpaired Student's *t*‐test; multiple group comparisons used one‐way or two‐way ANOVA with Tukey's or Sidak's post‐hoc test. Survival curves were analyzed by Kaplan‐Meier with log‐rank test. ROC curve analysis was performed, and the AUC with 95% confidence interval (CI) was calculated. Univariable and multivariable Cox regression analyses were performed to identify prognostic factors for PFS. Results are presented as hazard ratios (HRs) with 95% CIs. *P* < 0.05 was considered statistically significant. Additional methodological details are provided in Supplementary File S7.

## Author Contributions

WT, RQ and LY drafted the manuscript. WT, RQ and BA conducted in vitro and in vivo experiments. ZH, XM JJ, JL performed the analyses and interpreted all the data. LS, YZ, MJ, GX and RL prepared the figures and tables. YY and DX reviewed and revised the manuscript. All authors read and approved the final manuscript.

## Fundings

This work was supported by the National Natural Science Foundation of China (82373041,82503445) and Heilongjiang Province Natural Science Foundation (BS2025H008).

## Ethics Statement

The study protocol was approved by the Institutional Review Board of the hospital (Approval No. KY2023‐68), and written informed consent was obtained from all participants.

## Consent

Written consent was obtained from each participate.

## Conflicts of Interest

The authors declare no conflict of interest.

## Supporting information




**Supporting File 1**: advs75990‐sup‐0001‐SuppMat.docx.

**Supporting File 2**: advs75990‐sup‐0002‐TableS1.xlsx.

**Supporting File 3**: advs75990‐sup‐0003‐TableS2.xlsx.

**Supporting File 4**: advs75990‐sup‐0004‐TableS3.xlsx.

**Supporting File 5**: advs75990‐sup‐0005‐TableS4.xlsx.

**Supporting File 6**: advs75990‐sup‐0006‐TableS5.docx.

**Supporting File 7**: advs75990‐sup‐0007‐TableS6.docx.

**Supporting File 8**: advs75990‐sup‐0008‐FileS7.docx.

**Supporting File 9**: advs75990‐sup‐0009‐FileS8.docx.

## Data Availability

The data that support the findings of this study are available from the corresponding author upon reasonable request.
