# Supplementary material for: RBM10 Deficiency Promotes Anti‐PD‐1 Resistance in LUAD via STING Alternative Splicing‐Driven CCL7 Signaling and Macrophage Polarization
Source: Adv Sci (Weinh). 2026 Jun 22;13(49):e22159. doi: 10.1002/advs.202522159 (PMC13337095; doi:10.1002/advs.202522159)
Supplement: Supplementary file 1 [file ADVS-13-e22159-s001.docx]

**Figure S1. RBM10 orchestrates M2 polarization and infiltration in the LUAD TME.**

(A) Lollipop plot showing correlation between RBM10 expression and macrophage infiltration (M0, M1, M2) in TCGA-LUAD cohort, analyzed by multiple algorithms (n=515 patients). (B) Violin plots showing differences in M1 and M2 macrophage infiltration between RBM10-high and RBM10-low groups across algorithms. (C) WB validation of RBM10 knockdown in CMT167 cell line. (D) Subcutaneous tumor formation in C57BL/6 injected with shNC or shRBM10 LLC cells (n=6). (E-F) Subcutaneous tumor volume and weight at endpoint. (G-H) FC analysis of M1 (CD86⁺, Q1) and M2 (CD206⁺, Q3) macrophage infiltration in shNC or shRBM10 LLC (G) and CMT167 (H) subcutaneous tumors (n=5). All data are presented as the mean ± SEM (n ≥ 3). The *P* values in (B, F and H) were calculated using two-tailed unpaired Student’s t-test. The *P* values in panels (E) were calculated using two-way ANOVA. **P* < 0.05, ***P* < 0.01, ****P* < 0.001. Original blots can be found in Supplementary File 8.

**Figure S2. RBM10 in PC9 cells regulates macrophage recruitment and polarization in a tumor cell-macrophage co-culture system.**

(A) Schematic diagram of the tumor cell-macrophage co-culture system. [Created in BioRender. Gao, W. (2026) https://BioRender.com/awmu085] (B) WB validation of RBM10 transfection in PC9, 3255, and 292 cell lines. (C) FC analysis of CD68 expression in PMA-induced M0 macrophages (n=3). (D) qPCR analysis of M1 and M2 marker gene expression in macrophages co-cultured with PC9 shNC, sh1, and sh2 cells (n=3). (E-F) FC analysis of CD86 and CD206 surface expression on macrophages after co-culture (n=3). (G-H) Transwell assay of macrophage recruitment induced by CM from PC9 cells (n=3). (I-J) IF staining of CD86 and CD206 expression on macrophages after co-culture. (K) WB of CD86 and ARG1 expression in macrophages after co-culture. All data are presented as the mean ± SEM (n = 3). The *P* values in panels (C) were calculated using two-tailed unpaired Student’s t-test. The *P* values in panels (D, F, H-I) were calculated using one-way ANOVA. **P* < 0.05, ***P* < 0.01, ****P* < 0.001. Original blots can be found in Supplementary File 8.

**Figure S3. RBM10 in 3255 and 292 cells regulates macrophage recruitment and polarization in a tumor cell-macrophage co-culture system.**

(A-B) qPCR validation of RBM10 transfection in PC9, 3255 and 292 cell lines (n=3). (C) qPCR analysis of CD68 expression in PMA-induced M0 macrophages (n=3). (D) qPCR analysis of M1 and M2 marker genes in macrophages co-cultured with 292 vector and 292OE cells (n=3). (E, I) FC analysis of CD86 and CD206 surface expression on macrophages after co-culture with 292 vector and 292OE cells (n=3). (F) Transwell assay of macrophage recruitment induced by CM from 292 cells (n=3). (G) WB of CD86 and ARG1 expression in macrophages after co-culture. (H, J) IF staining of CD86 and CD206 on macrophages after co-culture. (K) WB of CD86 and ARG1 expression in macrophages after co-culture with 3255 shNC, sh1, and sh2 cells. (L) qPCR analysis of M1 and M2 marker gene expression in macrophages after co-culture (n=3). (M-N) Transwell assay of macrophage recruitment induced by CM from 3255 cells (n=3). All data are presented as the mean ± SEM (n = 3). The *P* values in panels (A, L and N) were calculated using one-way ANOVA. The *P* values in panels (B-D, F, I-J) were calculated using two-tailed unpaired Student’s t-test. ns (not significant), **P* < 0.05, ***P* < 0.01, ****P* < 0.001. Original blots can be found in Supplementary File 8.

**Figure S4. RBM10-mediated M2 macrophage reprogramming facilitates LUAD malignancy.**

(A) Schematic diagram of experimental design for evaluating macrophage effects on tumor cell phenotypes. [Created in BioRender. Gao, W. (2026) [https://BioRender.com/awmu085]](https://BioRender.com/awmu085%5d) (B, C) CCK-8 assay of PC9 and 292 cell proliferation treated with CM from co-culture groups (n=3). (D, E) Colony formation assay of PC9 and 292 cells treated with CM (n=3). (F, G) Wound healing assay of PC9 and 292 cell migration treated with CM (n=3). (H, I) Transwell migration and invasion assay of PC9 and 292 cells treated with CM (n=3). (J) Subcutaneous tumor formation in C57BL/6 mice bearing LLC shNC or shRBM10 tumors with or without macrophage depletion by clodronate liposomes (n=5). (K-L) Subcutaneous tumor volume and weight at endpoint. All data are presented as the mean ± SEM (n ≥ 3). The *P* values in panels (B-C) were calculated using two-way ANOVA. The *P* values in (D-I and K-L) were calculated using one-way ANOVA. ns (not significant), **P* < 0.05, ***P* < 0.01, ****P* < 0.001, *****P* < 0.0001.

**Figure S5. RBM10 regulates macrophage phenotype through CCL7-CCR2 axis.**

(A-D) GO and KEGG pathway enrichment analysis of differentially secreted cytokines from antibody array. (E-H) qPCR analysis of IL7 and OSM mRNA in PC9 and 292 cells (n=3). (I) qPCR analysis of CCR1, CCR2, and CCR3 mRNA expression in M0 macrophages under four conditions including A: M0 alone, B: M0 co-cultured with PC9 shNC cells, C: M0 co-cultured with PC9 shRBM10 cells, D: M0 treated with exogenous CCL7 (n=3). (J) FC analysis of CCR2 surface expression on macrophages across the A-D groups above mentioned (n=3). (K-L) Transwell assay of macrophage recruitment induced by CM from PC9 and 292 cells with CCR2 inhibitor, RS102895 (10 μM, 24 h) or CCL7 supplementation (100 ng/mL, 24 h) (n=3). All data are presented as the mean ± SEM (n =3). The *P* values in panels (F, H) were calculated using two-tailed unpaired Student’s t-test. The *P* values in panels (E, G, I and K-L) were calculated using one-way ANOVA. ns (not significant), **P* < 0.05, ***P* < 0.01, ****P* < 0.001, *****P* < 0.0001.

**Figure S6. RBM10 regulates macrophage phenotype through STING pathway.**

(A) KEGG pathway enrichment analysis of differentially expressed genes from RNA sequencing. (B) Quantification of mtDNA levels by IF in PC9 shNC and shRBM10 cells (n=3). (C-F) Transwell migration and invasion assay of PC9 and 292 cells treated with CM from co-cultures after H-151 (1 μM) or MSA-2 (5 μg/mL) treatment (n=3). All data are presented as the mean ± SEM (n = 3). The *P* values in panels (B) were calculated using two-tailed unpaired Student’s t-test. The *P* values in panels (E-F) were calculated using one-way ANOVA. ****P* < 0.001, *****P* < 0.0001.

**Figure S7. RBM10 regulates STING expression through its AS function.**

(A, B) Functional enrichment analysis of CLIP-seq data. (C, D) Number and types of AS events in PC9 shNC and shRBM10 cells based on CLIP-seq. (E) Schematic showing STING exon 3 as the most significant RBM10 binding region. (F) Functional domain of RBM10 binding to STING pre-mRNA. (G-H) ChIP assay of Pol II pSer2 binding to the STING promoter in cells with differential RBM10 expression (n=3). All data are presented as the mean ± SEM (n = 3). The *P* values in panels (G-H) were calculated using two-tailed unpaired Student’s t-test. ns (not significant), **P* < 0.05, *****P* < 0.0001.

**Figure S8. STING-E3(+) but not STING-E3(-) knockdown inhibits CCL7 secretion and M2 macrophage polarization.**

(A) WB analysis of RBM10 and STING expression in PC9 and 3255 cells: shNC, shRBM10, shRBM10+siNC, and shRBM10+siSTING-E3(+). (B) WB analysis of RBM10 and STING expression in 292 cells: vector, RBM10-OE, RBM10-OE+siNC, and RBM10-OE+siSTING-E3(-). (C-D) ELISA of CCL7 secretion in PC9, 3255 and 292 cells (n=3). (E-F) WB of CD86 and ARG1 in macrophages co-cultured with PC9, 3255 and 292 cells (n=3). (G-J) Transwell assay of macrophage recruitment induced by CM from PC9, 3255 and 292 cells (n=3). All data are presented as the mean ± SEM (n = 3). The *P* values in (C-D and I-J) were calculated using one-way ANOVA. **P* < 0.05, ****P* < 0.001, *****P* < 0.0001. Original blots can be found in Supplementary File 8.

**Figure S9. STING-E3(+) but not STING-E3(-) overexpression promotes CCL7 secretion and M2 macrophage polarization.**

(A) WB analysis of RBM10 and STING expression in PC9 and 3255 cells: vector, STING-E3(+)-1, STING-E3(+)-2. (B) WB analysis of RBM10 and STING expression in PC9 and 3255 cells: vector, STING-E3(-)-1, STING-E3(-)-2. (C-D) ELISA of CCL7 secretion in PC9 and 3255 cells (n=3). (E) WB of CD86 and ARG1 in macrophages co-cultured with PC9 and 3255 cells (n=3). (F-I) Transwell assay of macrophage recruitment induced by CM from PC9 and 3255 cells (n=3). All data are presented as the mean ± SEM (n = 3). The *P* values in (C, D, G, I) were calculated using one-way ANOVA. ns (not significant), **P* < 0.05, ****P* < 0.001, *****P* < 0.0001. Original blots can be found in Supplementary File 8.

**Figure S10. Inhibition of CCR2 or STING abrogates mitochondrial transfer in RBM10‑deficient tumor cells.**

(A) IF of MitoTracker-labeled mitochondrial transfer in PC9 and 3255 shRBM10 cells co-cultured with M2 macrophages treated with DMSO, RS102895 (10 μM) or H-151 (1 μM), respectively. (B-C) FC quantification of MFI in MitoTracker-labeled mitochondria transferred to co-cultured PC9 and 3255 shRBM10 cells treated with DMSO, RS102895 or H-151, respectively (n=3). (D-G) OCR measurements comparing basal OCR, spare respiratory capacity, proton leak and ATP production in PC9 and 3255 shRBM10 cells under co-culture conditions treated with DMSO, RS102895 or H-151, respectively (n=3). All data are presented as the mean ± SEM (n=3). The *P* values in panels (B, C, F and G) were calculated using two-tailed unpaired Student’s t-test. ns (not significant), **P* < 0.05, ***P* < 0.01, ****P* < 0.001.

**Figure S11 CCL7 inhibition potentiates anti-PD-1 therapy efficacy in RBM10-low LUAD**

(A-G) PD-L1 expression in PC9 and 292 cells under monoculture or co-culture with macrophages, assessed by qPCR (A-D), WB (E), and FC (F, G) (n=3). (H, I) FC analysis of PD-L1 expression (H) and CD3⁺CD8⁺ T cell infiltration (I) in subcutaneous shNC and shRBM10 LLC tumors. (J)Treatment schema for LLC shRBM10 tumor-bearing mice with shCCL7, anti-PD-1 antibody or not (n=5). [Created in BioRender. Gao, W. (2026) [https://BioRender.com/awmu085]](https://BioRender.com/awmu085%5d) (K) Subcutaneous tumor formation in C57BL/6 injected with shRBM10 LLC cells treated with shCCL7, anti-PD-1 antibody or not (n=5). (L) Tumor growth curves of indicated groups. (M) Survival curves of indicated groups. All data are presented as the mean ± SEM (n ≥ 3). The *P* values in panels (B, D, G, H and I) were calculated using two-tailed unpaired Student’s t-test. The *P* values in panels (A, C and F) were calculated using one-way ANOVA. The *P* values in panels (L) were calculated using two-way ANOVA. Survival curves (M) were calculated using log-rank test. ns (not significant), **P* < 0.05, ***P* < 0.01, ****P* < 0.001, *****P* < 0.0001. Original blots can be found in Supplementary File 8.

**Figure S12. The schematic representation of study.** Left panel (red): RBM10 proficiency promotes STING exon 3 skipping, resulting in decreased CCL7 secretion and reduced M2 macrophage polarization, thereby favoring immune activation (solid arrows). Right panel (blue): RBM10 deficiency promotes STING exon 3 inclusion, leading to enhanced CCL7 secretion and M2 macrophage polarization, thereby establishing an immunosuppressive microenvironment (solid arrows). M2 macrophages transfer mitochondria to tumor cells, potentially sustaining mtDNA-cGAS-STING signaling and CCL7 production, thus contributing to a positive feedback loop (dashed arrows). [Created in BioRender. Gao, W. (2026) [https://BioRender.com/awmu085]](https://BioRender.com/awmu085%5d)
