# Supplementary material for: RBM10 Deficiency Promotes Anti‐PD‐1 Resistance in LUAD via STING Alternative Splicing‐Driven CCL7 Signaling and Macrophage Polarization
Source: Adv Sci (Weinh). 2026 Jun 22;13(49):e22159. doi: 10.1002/advs.202522159 (PMC13337095; doi:10.1002/advs.202522159)
Supplement: Supplementary file 3 [file ADVS-13-e22159-s004.docx]

**Materials and Methods**

**Differentiation of macrophages**

THP-1 cells were seeded into 6-well plates at 1×10^6^ cells/well and treated with 100 ng/mL PMA (MCE, Shanghai) for 48h at 37°C under 5% CO₂ to induce differentiation into adherent macrophages.

**Establishment of co-culture System**

A transwell co-culture system (0.4 μm pore membrane; Corning, USA) was established with transfected LUAD cells (5×10^5^ cells/insert) in the upper chamber and PMA-differentiated THP-1 macrophages (1×10^6^ cells/well) in the lower chamber of a 6-well plate. Co-culture was maintained for 72h.

**Macrophage chemotaxis Assay**

Cell chemotaxis was assessed using 24-well Transwell chambers (Corning, USA). CM from RBM10-modified PC9, 3255, and 292 LUAD cells was mixed 1:1 with fresh medium and placed in the lower chambers. THP-1-derived macrophages (3×10^5^ cells) suspended in 200 μL serum-free medium were seeded into the upper inserts. After 48h of incubation, cells that migrated to the underside of the membrane were fixed, stained with crystal violet, and quantified using Image-Pro Plus 6.0.

**Cell migration and matrigel invasion Assay**

Cell migration and invasion were evaluated using 24-well Transwell chambers (Corning, USA). LUAD cells were treated with CM collected from co-cultures of RBM10-modified tumor cells and THP-1 macrophages. For migration assays, 1.2×10^4^ cells in 200 μL serum-free medium were placed in the upper chamber, with 800 μL complete medium in the lower chamber. For invasion, Matrigel-coated inserts (pre-coated with 40 μL Matrigel at 1:8 dilution) were used. After 48h of incubation, migrated/invaded cells were fixed, stained with crystal violet, and quantified with Image-Pro Plus 6.0.

**Wound healing Analysis**

90% confluent cells in 6-well plates were scratched using sterile pipette tips (100 μL). Migration progress was documented at baseline (0h) and 24h post-wounding, with wound closure rates calculated via Image-Pro Plus 6.0 image analysis.

**Cell proliferation assay**

Cell proliferation was evaluated using CCK-8 assays (Meilunbio, China). A total of 5×10^3^ cells per well were seeded in 96-well plates and treated with CM. CCK-8 reagent (10 μL/well) was added at indicated time points (0-72h), incubated for 1h at 37 °C, and absorbance was measured at 450 nm with ≥3 replicates. For colony formation, 800 cells/well were plated in 6-well plates. After 14 days, cells were fixed, stained with crystal violet, and colonies were quantified using Image-Pro Plus 6.0.

**Western blotting**

After collecting cells or LUAD tissues, proteins were extracted using RIPA lysis buffer (AR0102, BOSTER) supplemented with protease inhibitors including PMSF (ST506, Beyotime). Proteins were separated by SDS‑PAGE and transferred onto polyvinylidene difluoride (PVDF) membranes following standard protocols. The primary antibodies used are listed in **Supplementary Table 2**.

**Immunofluorescence staining**

Cells were incubated overnight at 4°C with primary antibodies (CD68, CD206, CD86, and dsDNA; **Supplementary Table 2**), followed by a 1h incubation at room temperature with Alexa Fluor 488- or 594-conjugated secondary antibodies. Nuclei were counterstained with DAPI before imaging by fluorescence microscopy.

**Cytokine antibody array and enzyme-linked immunosorbent assay (ELISA)**

Cytokine levels in 292 cell supernatants were measured using a human cytokine array (RayBiotech AAH-CYT-3), which simultaneously detects 42 cytokines. Following the manufacturer’s protocol, concentrated supernatants were incubated with antibody-coated membranes, followed by sequential incubation with biotinylated antibodies and HRP-streptavidin, and visualized by chemiluminescence. Signal intensities were quantified using ImageJ and analyzed with RayBio® software. Human CCL7 levels were further quantified using commercial ELISA kits (MeiMian, China), with absorbance measured at 450 nm.

**Chromatin immunoprecipitation (ChIP)**

CHIP was performed using the SimpleChIP® Enzymatic Chromatin IP Kit (Cell Signaling Technology, #9003) according to the manufacturer’s instructions. Immunoprecipitation of Pol II pSer2 on the STING chromatin was precipitated with an anti-RNA polymerase II antibody (Abcam, ab252855). Precipitated DNA was analyzed by qPCR, with results normalized to input DNA. ChIP-specific primers are listed in **Supplementary Table 3**.

**RNA-Seq and CLIP-Seq**

Total RNA was extracted from LUAD cells using TRIzol reagent (Invitrogen, USA) following the manufacturer’s instructions. mRNA was fragmented, reverse-transcribed, and amplified to construct cDNA libraries, which were then sequenced on an Illumina HiSeq X platform. For CLIP-seq, RNA-protein complexes were crosslinked and immunoprecipitated using an anti-RBM10 antibody (Sigma, HPA034972) with the CLIP Kit (IEMed, K319) in collaboration with IEMed Biomedical Technology (Guangzhou). Purified RNA fragments were subjected to high-throughput sequencing, followed by standard data analysis procedures.

**Agarose gel electrophoresis**

Splice variants were analyzed by electrophoretic separation of RT-PCR products on agarose gels stained with GelRed. Gels were visualized using a FluorChem E imaging system (ProteinSimple). Primer sequences are provided in **Supplementary Table 3**.

**RIP analysis**

PC9 and 292 cells at logarithmic growth phase were harvested and lysed at 4 °C for 1 h. Magnetic beads were pre-incubated with primary antibodies (anti-RBM10, HPA034972, Sigma; anti-QKI, 13169-1-AP, Proteintech) or control IgG for 2 h, followed by immunoprecipitation using a RIP Kit (Geneseed, China) according to the manufacturer’s protocol. RNA-protein complexes were then purified for downstream analysis. Primers used for RIP-qPCR are listed in **Supplementary Table 3**.

**RNA pull-down assay**

RNA-protein interactions were analyzed using the Pierce™ Magnetic RNA-Protein Pull-Down Kit (Thermo Fisher Scientific, USA) according to the manufacturer’s instructions, followed by WB detection of bound proteins.

**Proteomics analysis**

Proteomic analysis was conducted by Oebiotech. Total protein was extracted from PC9 and PC9-shRBM10 cells (n = 3 per group). Following quantification and quality control by SDS-PAGE, samples were digested with trypsin. The resulting peptides were desalted and analyzed by LC-MS/MS in data-independent acquisition (DIA) mode. Mass spectrometry data were processed for peptide identification, quantification, and statistical analysis.

**Mitochondrial transfer assay**

Macrophages were labeled with MitoTracker Red (Thermo Fisher Scientific, M7512) and then co-cultured with PC9 and 3255 cells for 48 h. Mitochondrial transfer was visualized using a Leica CD7 confocal microscope. For quantitative analysis, cells were washed with PBS and subjected to FC; MFI was determined using FlowJo software. For electron microscopy, PC9 cells were detached, washed in DPBS, and fixed in 2.5% glutaraldehyde for 3 h at room temperature. After storage in Sorensen buffer, samples were post-fixed with 2% osmium tetroxide for 1h. Ultrastructural analysis was performed using a Jeol JEM-1400 transmission electron microscope (Tokyo, Japan) operating at 80 kV.

**OCR measurements**

OCR was assessed with a Seahorse XFe96 Analyzer (Agilent). Cells were plated at 1×10^5^ cells/well and cultured to 70-90% confluency. After equilibrating for 1h in XF medium containing 10 mM glucose, 1 mM sodium pyruvate, and 2 mM glutamine in a non-CO₂ incubator, OCR was recorded at baseline and following sequential injection of oligomycin (1 μM), FCCP (1 μM), and rotenone/antimycin A (0.5 μM each). Data were normalized to protein content determined by the Pierce BCA assay.

**Cytosolic mtDNA extraction and quantification**

To quantify cytosolic mtDNA, cells were treated with EDTA-free trypsin (Thermo, 87785) and fractionated as described. mtDNA was extracted from the mitochondria-free cytosolic fraction using a DNA extraction kit (TianGen, DP304) and quantified via qPCR with mtDNA-specific primers (**Supplementary Table 3**). Equal volumes of DNA solution were used across all samples.

**Immunohistochemistry (IHC)**

IHC was performed on paraffin-embedded LUAD tissues and mouse tumor sections using antibodies in **Supplementary Table 2**. Staining results were evaluated by calculating a staining index (SI) based on both the proportion of positive cells scored as 0 for none, 1 for <10%, 2 for 10-35%, 3 for 35-75%, and 4 for ≥75% and staining intensity scored as 0 (negative), 1 (weak), 2 (moderate), and 3 (strong). The final SI was determined by multiplying these two scores (range 0-12), with SI ≥7 considered high expression and SI <7 considered low expression.

**Multiplex immuno-histochemistry (mIHC)**

Multiplex immunohistochemistry was performed on formalin-fixed, paraffin-embedded tissue sections using an iterative staining protocol. After deparaffinization, rehydration, and peroxidase blockade with 3% H₂O₂, heat-induced epitope retrieval was carried out in citrate buffer (95 °C, 5 min). Each staining cycle included incubation with a primary antibody (37 °C, 45 min), TBST washes, incubation with a fluorophore-conjugated secondary antibody (37 °C, 20 min), and additional washes. For exploratory mIHC analysis of immunotherapy response, pretreatment tumor biopsies from 8 patients within the 118-patient immunotherapy cohort were analyzed, including 4 responders (PR) and 4 non-responders (SD or PD) selected from cases with sufficient tissue for analysis. No marked differences were observed between the two groups in age (<65/≥65 years), sex, smoking status, tumor stage (III/IV), ECOG (0-1/2). Marker panels included RBM10, CD68, CD206, and CCR2 (Fig. 7A), or CD68, CD86, CD206, RBM10, and PD-L1 (Fig. 8A). Whole-slide images were acquired using a TissueFAXS SL system (v7.1.120) and analyzed with StrataQuest software (v7.1.129) for automated cell segmentation and spatial protein quantification.
