# Supplementary material for: RBM10 Deficiency Promotes Anti‐PD‐1 Resistance in LUAD via STING Alternative Splicing‐Driven CCL7 Signaling and Macrophage Polarization
Source: Adv Sci (Weinh). 2026 Jun 22;13(49):e22159. doi: 10.1002/advs.202522159 (PMC13337095; doi:10.1002/advs.202522159)
Supplement: Supplementary file 4 [file ADVS-13-e22159-s006.docx]

|  |  |  | RBM10 |  |  |
| --- | --- | --- | --- | --- | --- |
|  |  | high | low | total | *P* value |
| CD68 | high | 18 （41.9%） | 46（61.3%） | 64（54.2%） | 0.041 |
|  | low | 25（58.1%） | 29（38.7%） | 54（45.8%） |  |
|  | total | 43（36.4%） | 75（63.6%） | 118 (100%) |  |
| CD86 | high | 32 （74.4%） | 12（16%） | 44 （37.3%） | <0.001 |
|  | low | 11（25.6%） | 63（84%） | 74（62.7%） |  |
|  | total | 43（36.4%） | 75（63.6%） | 118 (100%) |  |
| CD206 | high | 16（37.2%） | 47（62.7%） | 63（53.4%） | 0.008 |
|  | low | 27（62.8%） | 28（37.3%） | 55（46.6%） |  |
|  | total | 43（36.4%） | 75（63.6%） | 118 (100%) |  |
| PD-L1 | high | 17（39.5%） | 44（58.7%） | 61（51.7%） | 0.045 |
|  | low | 26（60.5%） | 31（41.3%） | 57（48.3%） |  |
|  | total | 43（36.4%） | 75（63.6%） | 118 (100%) |  |

**Table S6. IHC staining of RBM10, CD68, CD86, CD206, and PD‑L1 in LUAD tissues from 118 patients receiving immunotherapy**
