# Supplementary material for: RBM10 Deficiency Promotes Anti‐PD‐1 Resistance in LUAD via STING Alternative Splicing‐Driven CCL7 Signaling and Macrophage Polarization
Source: Adv Sci (Weinh). 2026 Jun 22;13(49):e22159. doi: 10.1002/advs.202522159 (PMC13337095; doi:10.1002/advs.202522159)
Supplement: Supplementary file 5 [file ADVS-13-e22159-s009.docx]

**Table S5. Differentially expressed cytokines between 292 and 292 OE conditioned medium using a cytokine antibody array**

| Cytokines | 292 | 292OE | Fold change | Regulation |
| --- | --- | --- | --- | --- |
| Oncostatin M | 1987.166667 | 686.9423276 | 0.34601844 | Down |
| CCL7 | 122.6666667 | 42.07549008 | 0.348319327 | Down |
| IL-7 | 6353.666667 | 2475.041652 | 0.389641469 | Down |
| IL-2 | 113.6666667 | 55.80997647 | 0.495435841 | Down |
| Angiogenin | 8740.666667 | 4775.895135 | 0.546451302 | Down |
| TNF-beta | 211.6666667 | 117.2881537 | 0.556213889 | Down |
| GM-CSF | 1223.166667 | 684.326235 | 0.559830825 | Down |
| IGF-1 | 560.6666667 | 321.9974033 | 0.575069561 | Down |
| TNF-alpha | 1621.666667 | 996.2952832 | 0.614602681 | Down |
| TGF-beta 1 | 408.6666667 | 262.4812956 | 0.643160201 | Down |
| MIG | 727.6666667 | 473.7307769 | 0.651506098 | Down |
| VEGF | 7938.666667 | 5353.397587 | 0.67438569 | Down |
| IL-1beta | 226.6666667 | 172.2260993 | 0.760875985 | Down |
| MCP-2 | 163.1666667 | 125.1364316 | 0.768343746 | Down |
| MIP-1-delta | 249.6666667 | 195.1169099 | 0.782381289 | Down |
| IL-1alpha | 135.1666667 | 108.7858526 | 0.806260851 | Down |
| IL12-p40 | 168.1666667 | 136.9088485 | 0.81522472 | Down |
| IL-10 | 3581.666667 | 4404.409979 | 1.229645509 | UP |
| IL-8 | 62238.16667 | 81808.05118 | 1.314430375 | UP |
| SCF | 245.1666667 | 358.6227004 | 1.460891132 | UP |
| TARC | 186.1666667 | 273.5996894 | 1.467139925 | UP |
| MDC | 743.6666667 | 1463.267821 | 1.966339956 | UP |
| I-309 | 25.16666667 | 272.9456662 | 10.46926113 | UP |
