# Supplementary figures and images for: RBM10 Deficiency Promotes Anti‐PD‐1 Resistance in LUAD via STING Alternative Splicing‐Driven CCL7 Signaling and Macrophage Polarization
Source: Adv Sci (Weinh). 2026 Jun 22:e22159. Online ahead of print. doi: 10.1002/advs.202522159 (PMC13337095; doi:10.1002/advs.202522159)

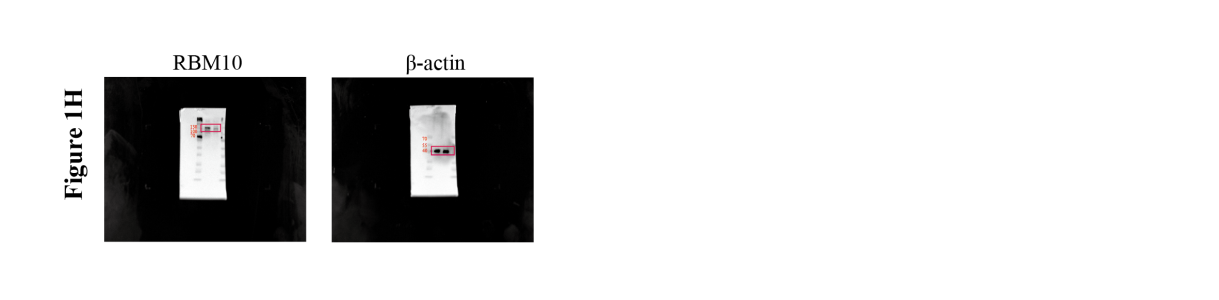

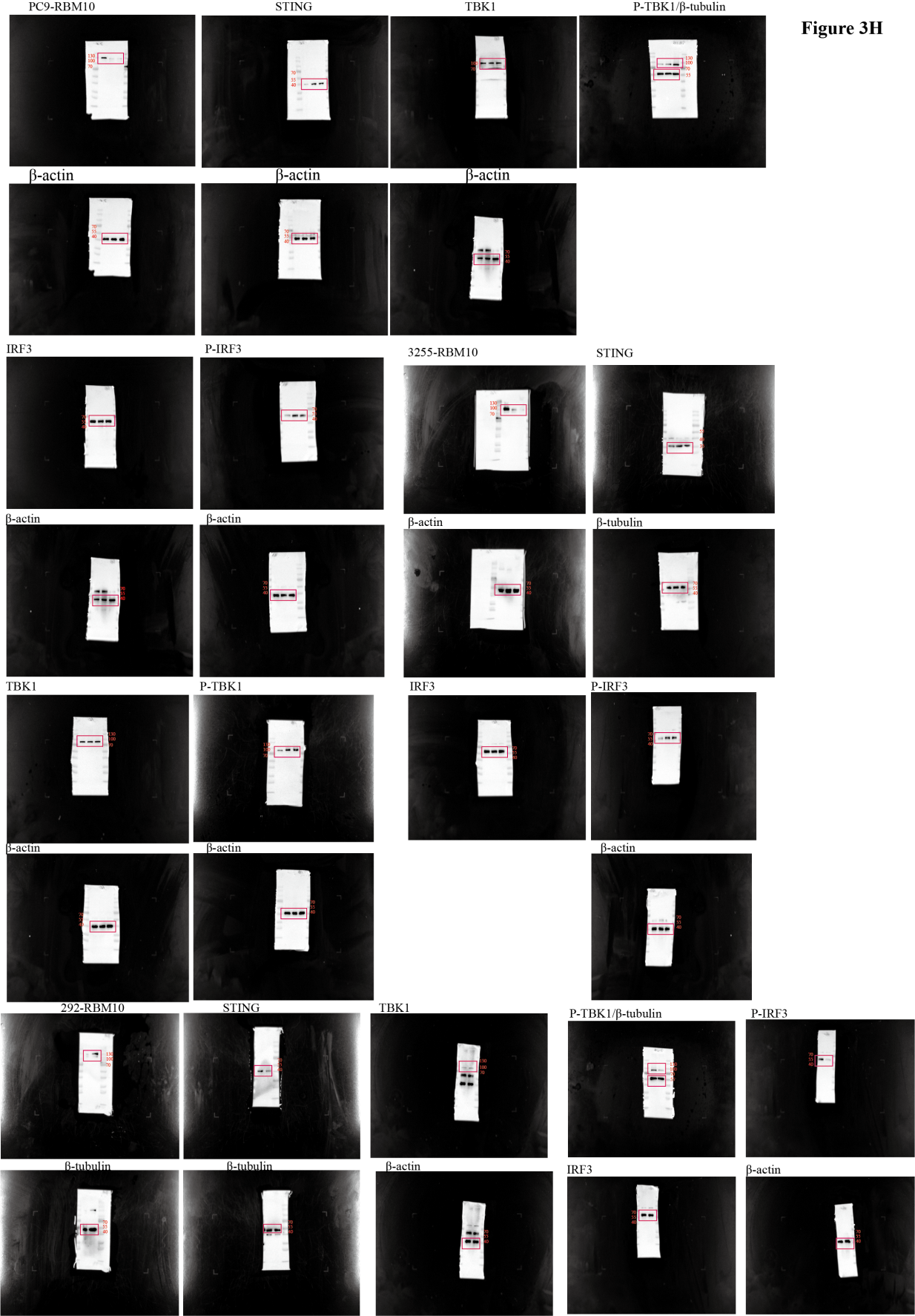

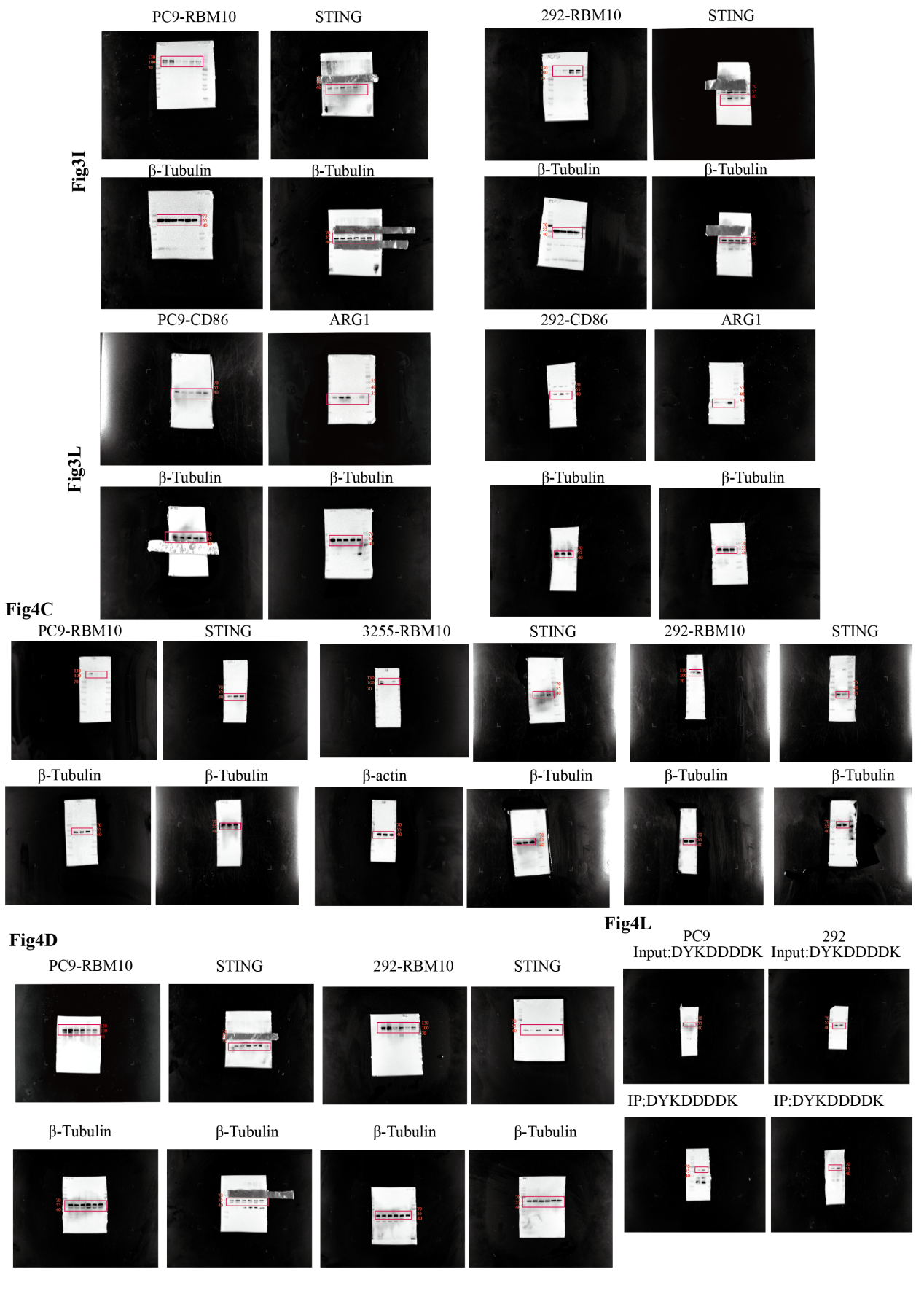


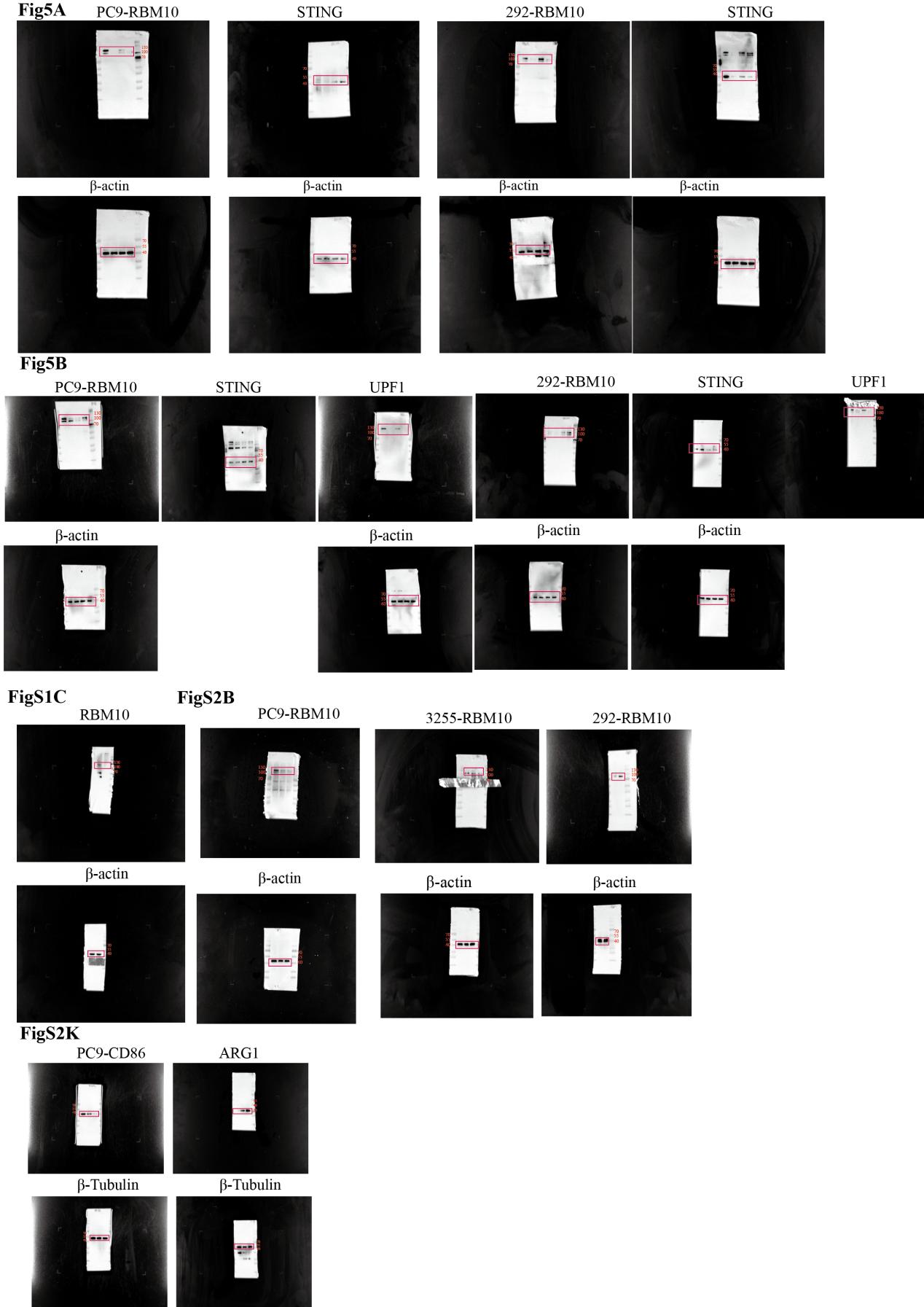


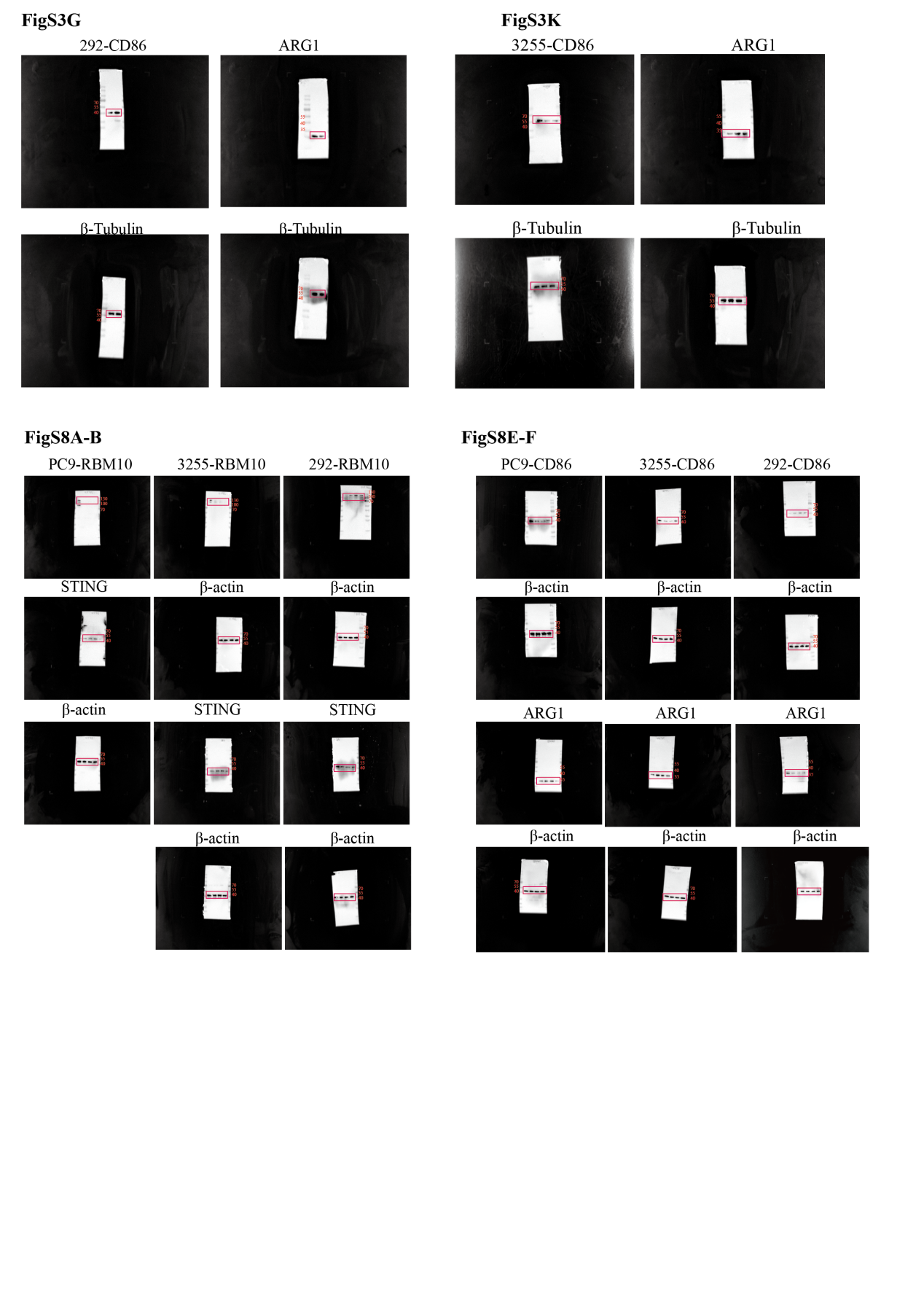


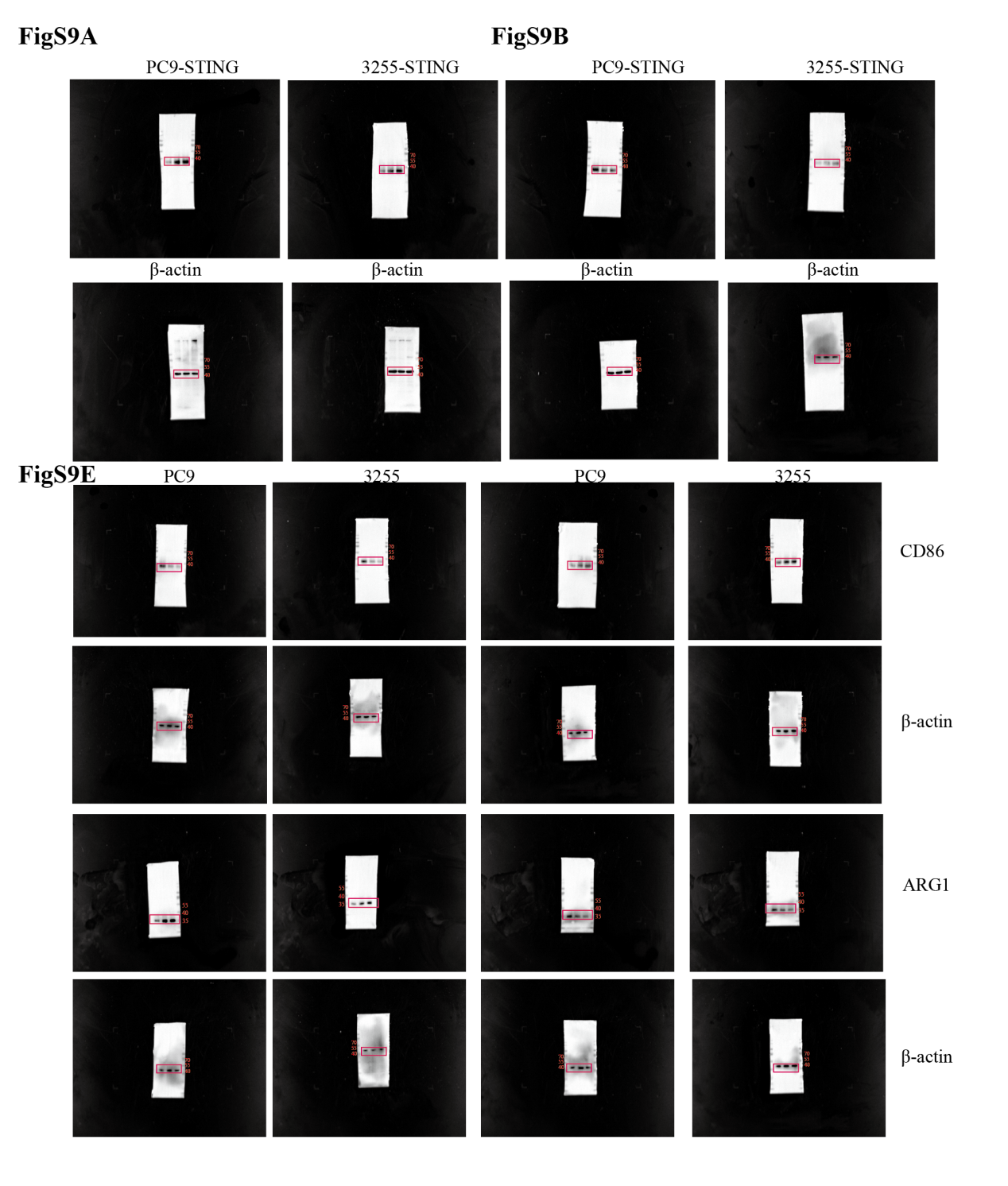


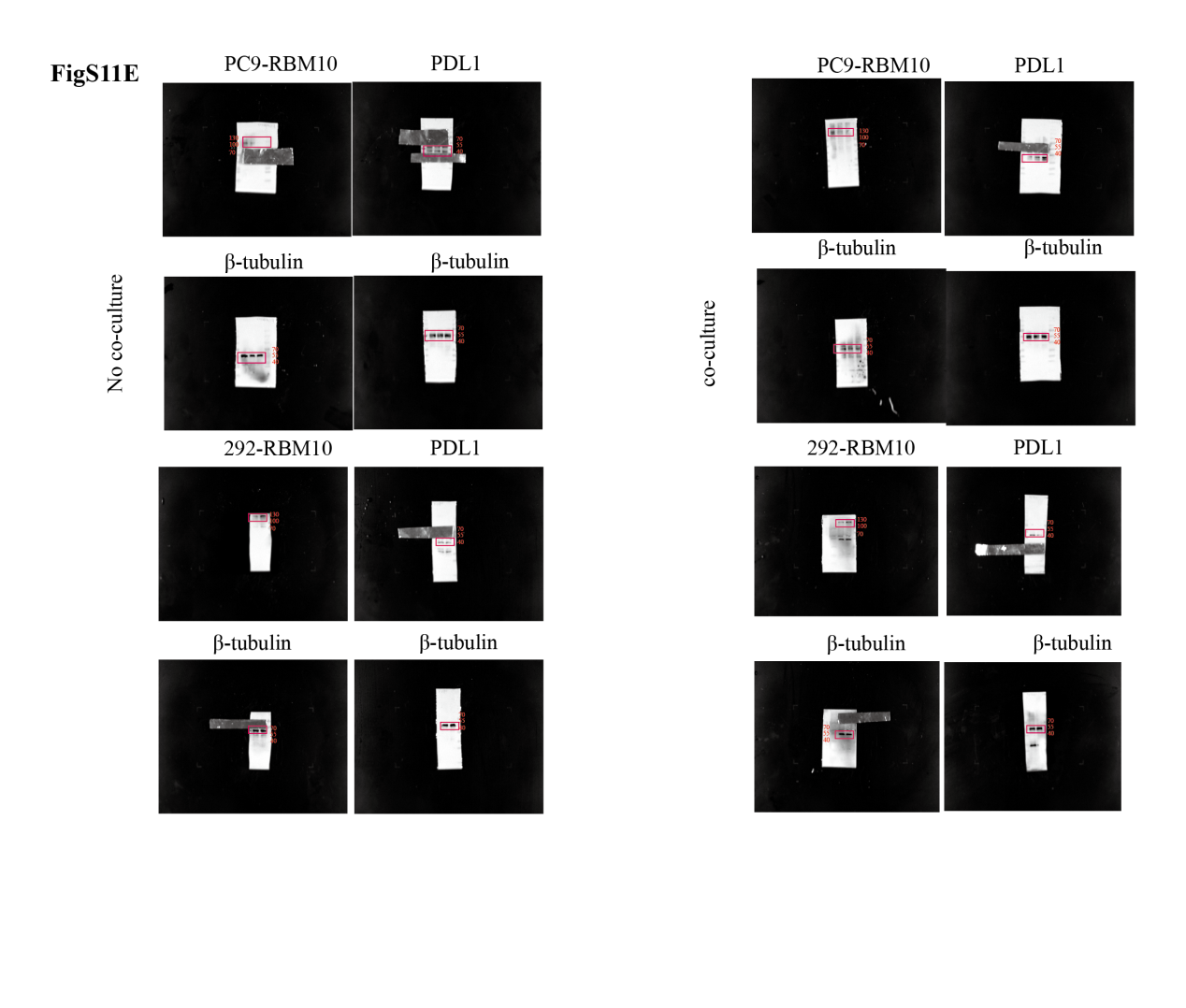


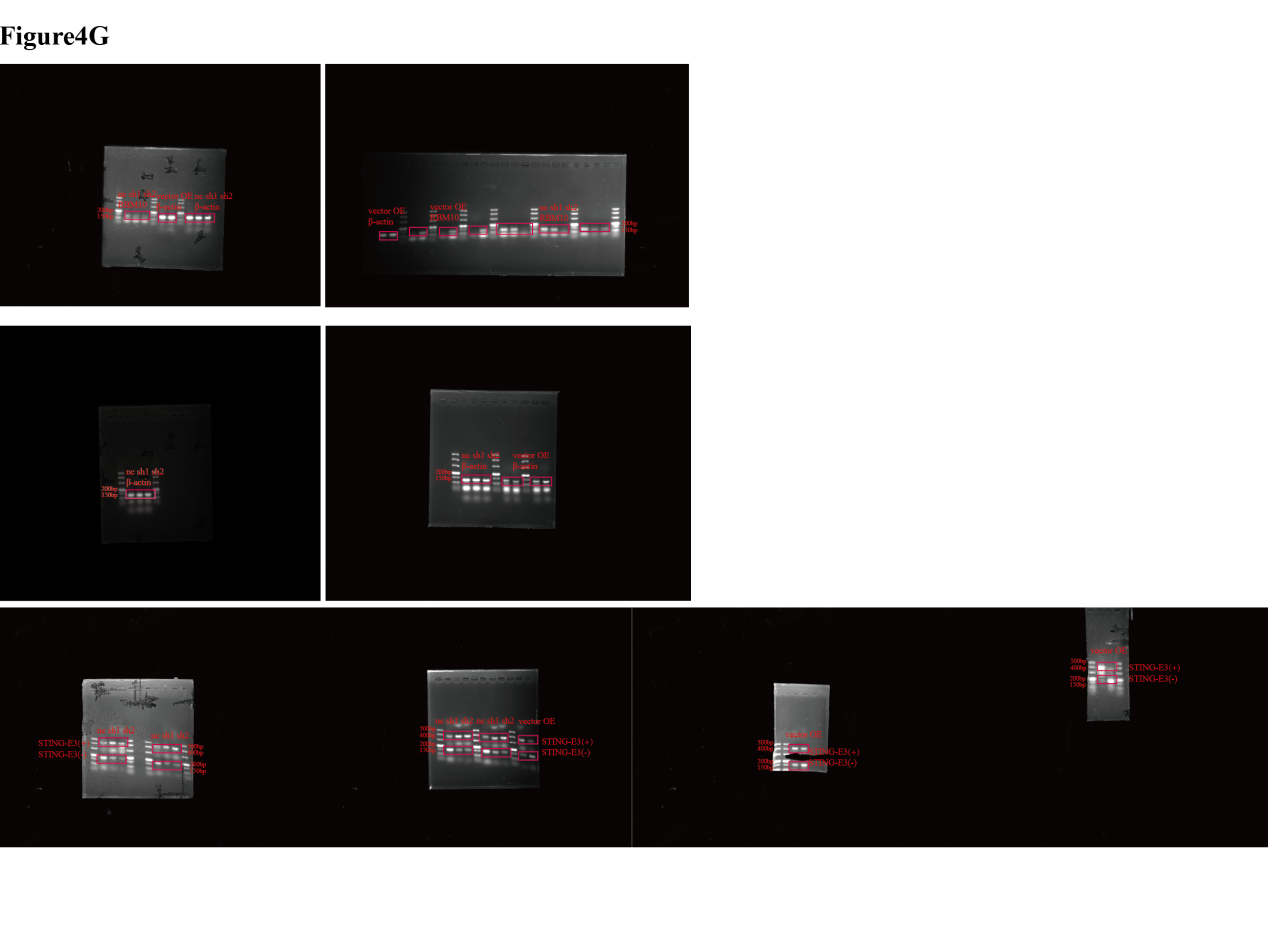

Supplement: Supplementary file 9 — Supporting File 9: advs75990‐sup‐0009‐FileS8.docx. [file ADVS-9999-e22159-s004.docx]
